# Inhibition of the NLRP3 inflammasome improves lifespan in animal murine model of Hutchinson–Gilford Progeria

**DOI:** 10.15252/emmm.202114012

**Published:** 2021-08-27

**Authors:** Alvaro González‐Dominguez, Raúl Montañez, Beatriz Castejón‐Vega, Jéssica Nuñez‐Vasco, Débora Lendines‐Cordero, Chun Wang, Gabriel Mbalaviele, José M Navarro‐Pando, Elísabet Alcocer‐Gómez, Mario D Cordero

**Affiliations:** ^1^ Instituto de Investigación e Innovación Biomédica de Cádiz INiBICA Hospital Universitario Puerta del Mar Cádiz Spain; ^2^ Institute of Molecular, Cell and Systems Biology University of Glasgow Glasgow UK; ^3^ Division of Bone and Mineral Diseases Washington University School of Medicine St. Louis MO USA; ^4^ Cátedra de Reproducción y Genética Humana del Instituto para el Estudio de la Biología de la Reproducción Humana (INEBIR) Universidad Europea del Atlántico (UNEATLANTICO)‐Fundación Universitaria Iberoamericana (FUNIBER) Seville Spain; ^5^ Departamento de Psicología Experimental Facultad de Psicología Universidad de Sevilla Sevilla Spain

**Keywords:** aging, NLRP3 inflammasome, progeria, Genetics, Gene Therapy & Genetic Disease, Immunology

## Abstract

Inflammation is a hallmark of aging and accelerated aging syndromes such as Hutchinson–Gilford progeria syndrome (HGPS). In this study, we present evidence of increased expression of the components of the NLRP3 inflammasome pathway in HGPS skin fibroblasts, an outcome that was associated with morphological changes of the nuclei of the cells. Lymphoblasts from HGPS patients also showed increased basal levels of NLRP3 and caspase 1. Consistent with these results, the expression of caspase 1 and Nlrp3, but not of the other inflammasome receptors was higher in the heart and liver of Zmpste24^−/−^ mice, which phenocopy the human disease. These data were further corroborated in Lmna^G609G/G609G^ mice, another HGPS animal model. We also showed that pharmacological inhibition of the NLRP3 inflammasome by its selective inhibitor, MCC950, improved cellular phenotype, significantly extended the lifespan of progeroid animals, and reduced inflammasome‐dependent inflammation. These findings suggest that inhibition of the NLRP3 inflammasome is a potential therapeutic approach for the treatment of HGPS.

## Graphical abstract

This study reveals a critical role of the NLRP3‐inflammasome complex in the pathogenesis of Hutchinson‐Gilford Progeria (HGPS). Targeting NLRP3 may be a novel potential therapeutic strategy for progeria treatment.

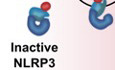

**The paper explained**

**Problem**

Progeria is a very rare disease associated to a fatal, pediatric autosomal dominant premature aging disease caused by a mutation in the LMNA gene. This mutation results in accumulation of a high level of an aberrant protein, termed progerin, which is accumulated in many tissues and is responsible for the diverse pathophysiological events and rapid degeneration. Children die predominantly from premature atherosclerotic cardiovascular disease. However, no specific therapeutic target has been defined.

**Results**

Our findings show NLRP3 inflammasome complex activation in cells from patients. Skin fibroblasts and lymphoblasts from HGPS syndrome showed high levels of NLRP3, caspase 1, and IL‐1β. Similar findings were observed in HGPS animal models in heart and liver. Interestingly, pharmacological inhibition of MCC950 using MCC950 increased survival and bodyweight and reduced IL‐1β levels of animal model of HGPS.

**Impact**

Our data suggest an essential role for NLRP3 signaling in the pathogenesis of HGPS and reveal a promising therapeutic target for progeria treatment.

## Introduction

Aging is associated with progressive impairment of homeostasis at the cell, tissue, and organismal level (López‐Otín *et al*, 2013). Aging is also associated with inflammation, a process known as inflamm‐aging during which key inflammatory pathways such as the NLRP3 inflammasome are activated (Franceschi *et al*, 2006).

The NLRP3 inflammasome is one of the most well‐studied inflammasomes in humans and mice (Afonina *et al*, 2017). It is a multiprotein complex comprising NLRP3 itself as an intracellular sensor, the adapter protein ASC, and the catalytic subunit, caspase 1. The NLRP3 inflammasome is activated by a range of danger and stress signals (Afonina *et al*, 2017) some of which arise during aging (Cordero *et al*, 2018). Consistent with this view, ablation of *Nlrp3* in mice has been shown to improve lifespan and healthspan by attenuating multiple age‐related degenerative changes such as cardiac aging, insulin sensitivity, bone loss, and ovarian aging (Youm *et al*, 2013; Marín‐Aguilar *et al*, 2020; Navarro‐Pando *et al*, 2021). However, the role of the NLRP3 inflammasome has not been assessed in genetic models of accelerated aging such as Hutchinson–Gilford progeria syndrome (HGPS), a rare premature aging condition in which a point mutation in the LMNA gene (c.1824C>T; GGC>GCT; p.G608G) (Schreiber & Kennedy, 2013) causes the accumulation of aberrant lamin A precursor of lamin A at the nuclear envelope, resulting in the disruption of the nuclear membrane architecture, abnormal gene transcription, and signal transduction. The clinical phenotype is characterized by delayed loss of primary teeth, alopecia, osteoporosis, abnormal skin pigmentation, accelerated cardiovascular disease, growth impairment, lipodystrophy, dermal abnormalities, and metabolic alterations (Lai & Wong, 2020). Nuclear factor κB (NF‐κB) mediates the secretion of high levels of pro‐inflammatory cytokines in the mouse HGPS model caused by the absence of Zmpste24, the integral membrane zinc metalloprotease involved in the proteolytic processing of farnesylated prelamin‐A, the precursor of the nuclear scaffold protein lamin A (Osorio *et al*, 2012). Accordingly, genetic and pharmacological inhibition of NF‐κB signaling prevents age‐associated features in these animal models and extends their longevity (Osorio *et al*, 2012). Interestingly, NF‐κB is a central mediator of NLRP3 inflammasome priming signals (Elliott & Sutterwala, 2015). In the present work, we report that human skin fibroblasts and lymphoblasts from patients with HGPS and Zmpste24^−/−^ mice exhibited increased activation of the NLRP3 inflammasome, a response that was corroborated by Lmna^G609G/G609G^. In addition, we showed that this alteration is detrimental as patients' skin fibroblasts and Zmpste24^−/−^ mice treated with MCC950, a specific inhibitor of NLRP3, showed improved phenotype evidenced by the amelioration of progeroid features (e.g., decreased inflammation and extended longevity) compared to untreated Zmpste24^−/−^ mice.

## Results

### HGPS patients' cells exhibit hyperactivated NLRP3 inflammasome

**Figure 1:**
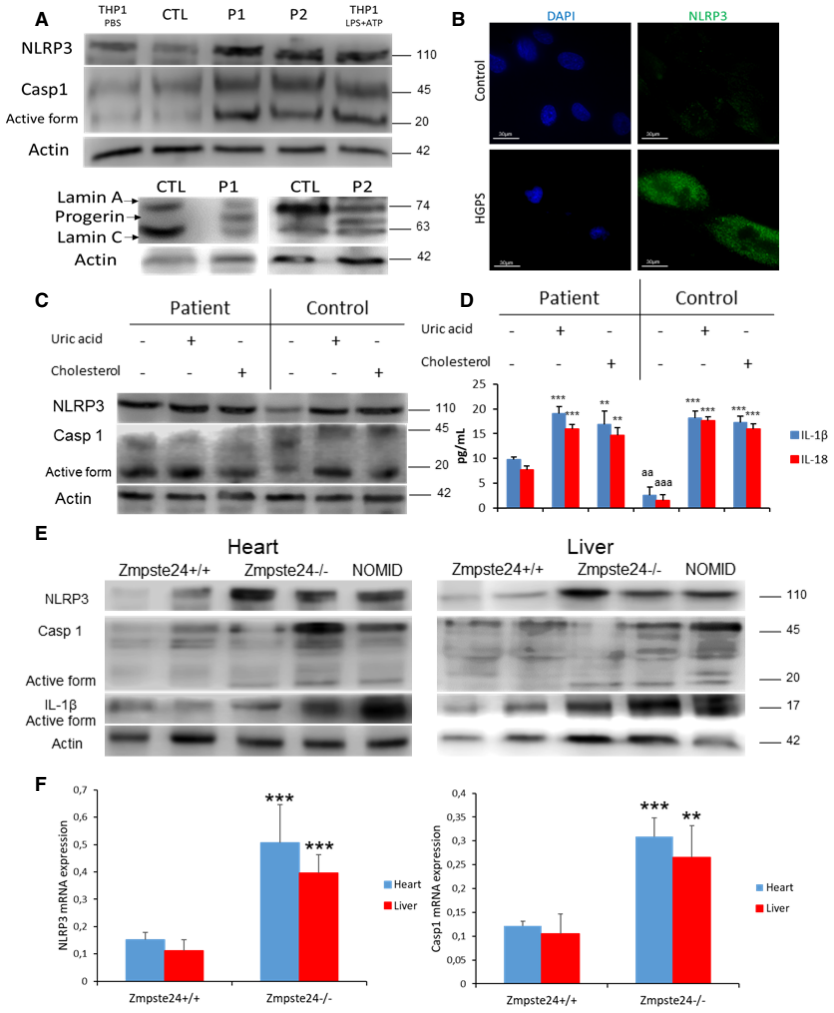
NLRP3 signaling is associated with HGPS Western blot analysis with representative blot including lamin A/C, NLRP3, caspase 1, and actin levels in skin fibroblasts from patients with HGPS, *n* = 2 controls and 2 patients. Positive control correspond to THP1 cells stimulated with LPS+ATP.Immunofluorescence (IF) visualization of NLRP3 (green) and nuclei (blue) in skin fibroblasts from a representative patient and control. Scale bar: 30 µm.Protein expression of NLRP3 and caspase 1 in lymphoblasts from control and one patient after stimulation with uric acid and cholesterol crystal.IL‐1β and IL‐18 medium release from lymphoblasts which were assessed after a 24‐h incubation with uric acid and cholesterol. ***P < 0.001, **P < 0.005, * treatment vs no treatment*;* ^aaa^ *P* < 0.001; ^aa^ *P* < 0.01 control cells vs patient cells.Western blot analysis with representative blot including NLRP3, caspase 1, IL‐1β and actin levels in heart and liver tissues from wild‐type and Zmpste24^−/−^ mice.NLRP3 and caspase 1 transcript expression levels were determined in heart and liver tissues by real‐time quantitative RT–PCR. *n* = 5 for Zmpste24^+/+^ and *n* = 5 for Zmpste24^−/−^ groups respectively. ***P < 0.001, **P < 0.005, wild‐type vs Zmpste24^−/−^ mice. Data are showed means ± SD, *n* = 4 mice per group. Data information: Results are presented as the mean ± SD of three independent experiments. One‐way ANOVA test was used for statistical analysis Source data are available online for this figure.

To evaluate the role of the NLRP3 inflammasome in progeria, we examined the expression of the components of the NLRP3 inflammasome pathway in HGPS and control fibroblasts. THP1 cells treated with LPS + ATP were used as positive control. Immunoblotting analysis showed higher expression of NLRP3 and capsase‐1 in HGPS fibroblasts (Fig 1A), data that were corroborated by immunofluorescence (Fig 1B).

It is known that soluble circulating factors can induce cardiac and metabolic damage and systemic inflammation (Libby *et al*, 2016). Furthermore, metabolic alterations have been observed in animal models of HGPS with lipid accumulation (Mariño *et al*, 2008). To evaluate the response of the NLRP3 inflammasome complex to metabolic alterations, we exposed HGPS lymphoblasts and age‐ and passage‐matched controls to cholesterol crystals and uric acid, two well‐known metabolic inducers of this inflammasome (López‐Otín *et al*, 2013; Cordero *et al*, 2018). HGPS cells showed increased basal levels of NLRP3 and caspase 1 protein expression with a moderate increase after exposure to cholesterol crystals and uric acid compared to control cells (Fig 1C). This response was accompanied by increased IL‐1β and IL‐18 release in naïve condition compared with control and similar release than control after cholesterol and uric acid treatment (Fig 1D).

### Zmpste24‐deficient mice exhibit hyperactivated NLRP3 inflammasome

**Figure EV1:**
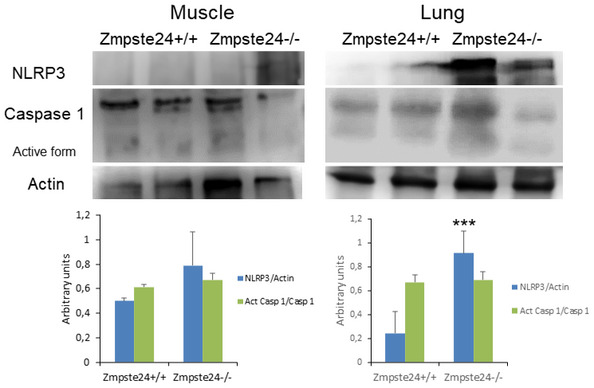
NLRP3 inflammasomes expression in lung and muscle from progeroid animals Western blot analysis with representative blot including NLRP3, caspase 1, and actin levels in lung and muscle tissues from wild‐type and Zmpste24^−/−^ mice. Densitometric analysis is shown as means ± SD, *n* = 5 mice per group. Data are shown as means ± SD. ***P < 0.001, wild‐type vs Zmpste24^−/−^ mice. One‐way ANOVA test was used for statistical analysis. Source data are available online for this figure.

**Figure EV2:**
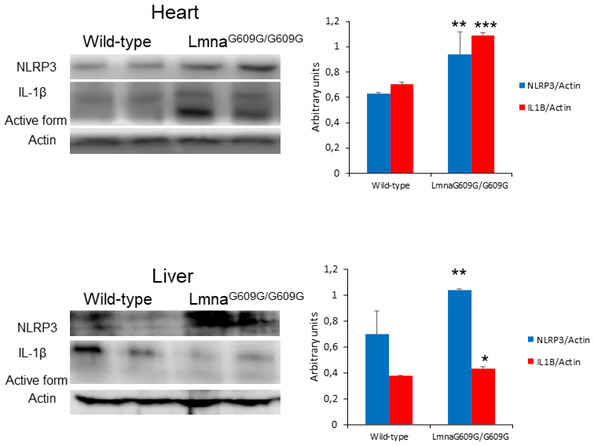
NLRP3 inflammasomes expression in heart and liver from Lmna^G609G/G609G^ mice Western blot analysis with representative blot including NLRP3, IL‐1β, and actin levels in cardiac and liver tissues from wild‐type and Lmna^G609G/G609G^ mice. Densitometric analysis is shown as means ± SD, *n* = 4 mice per group. Data are shown as means ± SD. ***P < 0.001, **P < 0.005, *P < 0.05 wild‐type vs Lmna^G609G/G609G^ mice. One‐way ANOVA test was used for statistical analysis. Source data are available online for this figure.

To extend *in vitro* observations to an *in vivo* setting, we evaluated the inflammasome complex status in a murine model of progeria. For this purpose, we used a mouse model in which the absence of Zmpste24 metalloproteinase leads to a progeroid phenotype similar to the human premature aging syndrome. Examinations of basal levels of inflammasome components revealed higher expression of Nlrp3, caspase 1 and IL‐1β mRNAs, and proteins in heart and liver tissues (Fig 1E and F and Appendix Fig S1 and S2), but not in muscle (Fig EV1) of Zmpste24^−/−^ mice compared to wild‐type animals. Furthermore, the NLRP3 inflammasome was also activated in the heart and moderately in liver in the Lamin^G609G/G609G^ mouse model (Fig EV2). These new findings reveal different effects of the inflammasomes in two genetic different models of HGPS, preliminary observations that will be further investigated.

### Inhibition of the NLRP3 inflammasome improves the progeria phenotype

**Figure 2:**
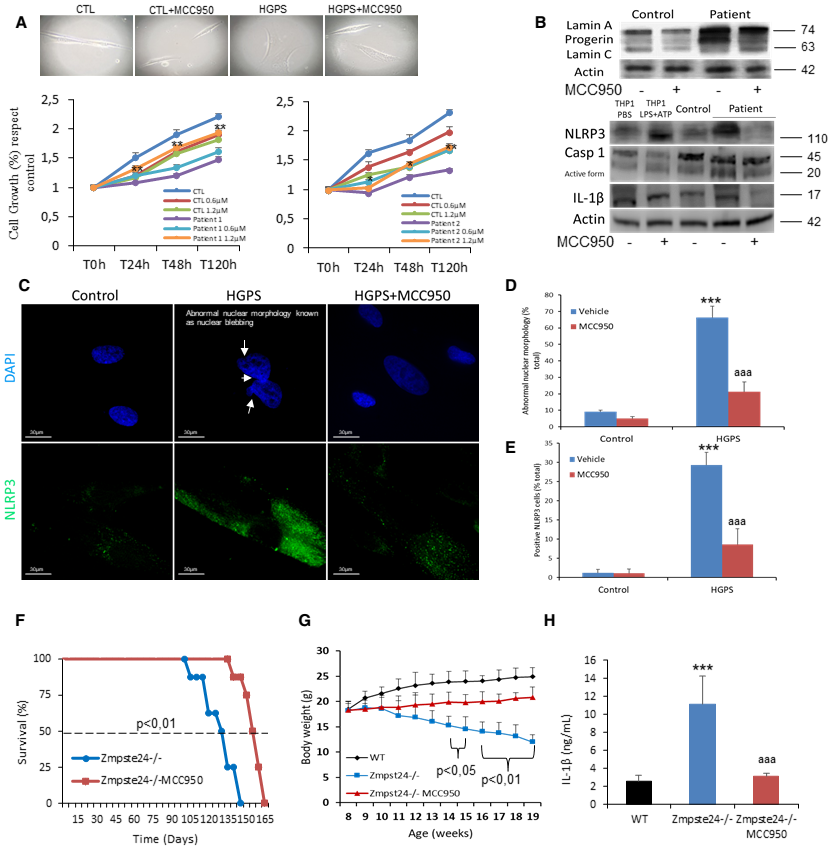
NLRP3 inhibition by MCC950 ameliorates skin fibroblasts and Zmpste24‐deficient mice progeroid phenotypes ACell growth (lower subpanel left patient 1 and right patient 2) and morphological aspect (upper subpanel) with MCC950 determined in healthy and representative HGPS fibroblasts. **P < 0.01, *P < 0.05 patient cells no treatment vs treatment. Results are presented as the mean ± SD of three independent experiments.BWestern blot analysis with representative blots including lamin A/C, NLRP3, IL‐1β, and actin levels in skin fibroblasts from control and HGPS patient after 48 h of vehicle and MCC950 treatment.C–ERepresentative fluorescence images of HGPS and control fibroblasts to evaluate the effect of the MCC950 in the nuclear morphology (D) and NLRP3 expression (E). Scale bar: 30 µm. Results are presented as the mean ± SD of three independent experiments. ***P < 0.01 patient vs control cells, ^aaa^ *P* < 0.01 patient cells no treatment vs treatment. One‐way ANOVA test was used for statistical analysis.FKaplan–Meier graph showing a significant increase in the maximum lifespan in WT mice compared with Zmpste24^−/−^ mice. *N* = 7 per group.GBody weights evolution of the groups over time. Data are showed means ± SD, *n* = 6 mice per group.HAnalysis of serum concentrations of IL‐1β measured by ELISA. *N* = 6 per group. Data are shown as means ± SD. ***P < 0.001, wild‐type vs Zmpste24^−/−^ mice; ^aaa^ *P* < 0.001; vehicle vs MCC950. Source data are available online for this figure.

To examine whether pharmacological inhibition of NLRP3 could be an effective treatment in HGPS, we assessed the effect of MCC950 on mutant fibroblasts. Control and HGPS patients' fibroblasts were treated at two different concentrations of MCC950 (0.6 and 1.2 µM). The results showed a statistically significant dose‐dependent increase in the growth rate of patient fibroblasts (Fig 2A). Western blot analyses showed that MCC950 reduced progerin signals in HGPS cells, responses that were associated with reduced NLRP3, caspase 1, and IL‐1β expression (Fig 2B, Appendix Fig S3 and S4). Interestingly, inhibition of the NLRP3 inflammasome expression and activity by MCC950 reduced the frequency of abnormal nuclear morphology in both control and HGPS fibroblasts (Fig 2C–E).

### Inhibition of the NLRP3 inflammasome extends the longevity of Zmpste24‐deficient mice

We explored *in vivo* effects of pharmacological inhibition of NLRP3 in Zmpste24^−/−^ mice. MCC950 was administered by i.p. route at 20 mg/kg daily. We found that treatment with MCC950 resulted in a significantly extended longevity of Zmpste24^−/−^ mice, with a mean increase lifespan of 19.2% (*P* < 0.01), an improvement in body weight from 14.7 ± 0.7 to 18.6 ± 0.7 g (*P* < 0.001), and a reduction in IL‐1β production (Fig 2F–H).

## Discussion

Genetic deletion of Nlrp3 in mice has been shown to improve lifespan and healthspan by attenuating multiple age‐related degenerative changes such as cardiac aging, insulin sensitivity with glycemic control, bone loss, cognitive function, and motor performance (Youm *et al*, 2013; Cordero *et al*, 2018; Marín‐Aguilar *et al*, 2020). Cardiovascular diseases are reduced in NLRP3 deficient mice as NLRP3‐assembled inflammasome is up‐regulated in atherosclerosis, myocardial infarction, ischemic heart disease, chronic heart failure, and hypertension (Marín‐Aguilar *et al*, 2020). Cardiovascular diseases have also been shown to be accelerated in HGPS patients and progerin. Furthermore, the abnormal form of prelamin‐A has also been shown to induce atherosclerosis and cardiac electrophysiological alterations (Hamczyk *et al*, 2018) and exogenously expressed progerin was shown to increase inflammation. Finally, a significant correlation was observed between inflammation and ZMPSTE24, and abnormal lamin A/C expression associated with progerin levels was observed in cardiovascular patients (Bidault *et al*, 2020; Messner *et al*, 2020). These changes altered the morphology of the nucleus, and progerin has been shown to induce IL‐1β (Bidault *et al*, 2020). Our results suggest that progerin was responsible for the activation of the NLRP3 inflammasome complex, which was associated with alterations of nuclear morphology. On the other hand, the AIM2 inflammasome has been shown to be activated after pharmacological alterations of the nuclear envelope integrity compatible with laminopathies (Di Micco *et al*, 2016). Thus, it is tempting to speculate that inflammasomes contribute to increased aging observed in progeroid syndromes.

Efforts have been launched to find specific pharmacological inhibitors of NLRP3. While several of these inhibitors have been shown to have a specific direct action on NLRP3, others have been shown indirect inhibitory effects (Zahid *et al*, 2019). MCC950 and analogs are selective small molecule inhibitors of the NLRP3 inflammasome, with remarkable therapeutic potential in human diseases. A few anti‐inflammatory strategies for the treatment of HGPS have been poorly explored (Lai & Wong, 2020) though some of the drugs that treat HGPS such as metformin, resveratrol, rapamycin, quercetin or spermidine indirectly inhibit inflammatory pathways such as the NLRP3 inflammasome (Cordero *et al*, 2018). In the current study, we provided evidence showing that the NLRP3 inflammasome complex is an important determinant of HGPS in human cells and mice. We showed that the specific expression of NLRP3 and other components of the complex rapidly increased in the skin fibroblasts from patients and in specific tissues associated with the progeroid phenotype and associated with inflammation such as heart or liver (Osorio *et al*, 2012; Schreiber & Kennedy, 2013; Lai & Wong, 2020). Moreover, we found continuous activation of the NLRP3 inflammasome in immune cells such as lymphocytes from patients. Finally, pharmacological inhibition of the NLRP3 inflammasome by MCC950 improved cell survival and morphology, reduced inflammation, and resulted in extended longevity of HGPS mice. Interestingly, we found that MCC950 treatment reduced the protein levels of NLRP3 in HGPS. These data could not be associated with the described mechanism of MCC950, which binds to NLRP3 and blocks its ability to hydrolyze ATP, and thus prevents it from maintaining its active structural conformation, and inhibiting NLRP3‐induced ASC oligomerization and reducing cleavage of caspase 1 (Coll *et al*, 2019; Tapia‐Abellán *et al*, 2019). However, previous data have showed similar effect of MCC950 about the inhibition of NLRP3 protein (Mridha *et al*, 2017; Gordon *et al*, 2018). These data could show the effect of MCC950 on the priming step of NLRP3 activation. Therefore, this HGPS treatment strategy, focused on the inhibition of NLRP3 inflammasome complex, could constitute an alternative therapy to slow down disease progression in patients with progeria.

## Materials and Methods

### Reagents

Trypsin was purchased from Sigma Chemical Co., (St. Louis, Missouri). Anti‐actin monoclonal antibody from Calbiochem‐Merck Chemicals Ltd. (Nottingham, UK). Lamin A/C, NLRP3 and caspase 1 were obtained from Cell Signaling Technology. NLRP3 inhibitors MCC950 was obtained from Sigma‐Aldrich (Saint Louis, USA). A cocktail of protease inhibitors (complete cocktail) was purchased from Boehringer Mannheim (Indianapolis, IN). Grace's insect medium was purchased from Gibco. The Immun Star HRP substrate kit was from Bio‐Rad Laboratories Inc. (Hercules, CA).

### Fibroblast culture

All fibroblasts from patients with HGPS were obtained from The Progeria Research Foundation Cell and Tissue Bank (http://www.progeriaresearch.org). An informed consent was obtained from all human subjects, and the experiments were done conformed to the WMA Declaration of Helsinki and to the principles set out in the Department of Health and Human Services Belmont Report. The following fibroblasts were used: HGADFN367 (3‐year‐old male) and HGADFN155 (1.2‐year‐old female). Control fibroblasts was used the HGADFN368 (37‐year‐old female), mother of the HGADFN367. Fibroblasts were cultured in high glucose Dulbecco's modified media (; Gibco, Invitrogen, Eugene, OR, USA) supplemented with 15% fetal bovine serum (FBS; Gibco, Invitrogen, Eugene, OR, USA), 1% GlutaMAX (Thermo Fisher) and antibiotics (Sigma Chemical Co., St. Louis, MO, USA). Cells were incubated at 37°C in a 5% CO_2_ atmosphere. The medium was changed every 2 days to avoid changes in pH.

We also used the following lymphoblasts: HGALBV009 (5.1‐year‐old male) and HGALBV021 (father of the proband, 37‐year‐old). Lymphoblasts were cultured in RPMI‐1640 (Gibco, Invitrogen, Eugene, OR, USA) supplemented with 15% FBS (Gibco, Invitrogen, Eugene, OR, USA), and antibiotics (Sigma Chemical Co., St. Louis, MO, USA). Cells were incubated at 37°C in a 5% CO_2_ atmosphere.

As a positive control of the NLRP3 inflammasome complex activation, we used THP1 cells primed with 200 ng/ml ultrapure LPS for 4 h, followed by stimulation with PBS (mock), 5 mM ATP (30 min).

### Real‐time quantitative PCR

The expression of the NLRP3 and caspase 1 genes was analyzed by SYBR Green quantitative PCR of mRNA extracted from GCs according to the previously described methodology (Marín‐Aguilar *et al*, 2020). Briefly, total cellular RNA was purified from the cells by the Trisure method (Bioline, London, UK). RNA concentration was determined spectrophotometrically. RNA samples were subsequently reverse transcribed to cDNA using the QuantiTect Reverse Transcription Kit (Qiagen, Hilden, Germany). PCR amplifications were conducted with primers targeting NLRP3 (NM_004895.4) and beta‐actin, used as an internal control. Thermal cycling conditions used were denaturation at 95°C for 20 s, 40 cycles of priming at 54°C for 20 s, and elongation at 72°C for 20 s. All reactions were performed in duplicate, and reaction mixes without RNA were used as negative controls in each run. Absence of contaminating genomic DNA was confirmed by setting up control reactions with RNA that had not been reverse transcribed. Fold changes in the expression of genes of interest were calculated using the ΔΔCt method.

### Western blotting

Whole cellular lysate from fibroblasts was prepared by gentle shaking with a buffer containing 0.9% NaCl, 20 mM Tris‐HCl, pH 7.6, 0.1% Triton X‐100, 1 mM phenylmethylsulfonylfluoride and 0.01% leupeptin. The protein content was determined by the Bradford method. Electrophoresis was carried out in a 10–15% acrylamide SDS–PAGE, and proteins were transferred to Immobilon membranes (Amersham Pharmacia, Piscataway, NJ). Next, membranes were washed with PBS, blocked over night at 4°C, and incubated with the respective primary antibody solution (1:1,000). Membranes were then probed with their respective secondary antibody (1:2,500). Immunolabeled proteins were detected by chemiluminescence method (Immun Star HRP substrate kit, Bio‐Rad Laboratories Inc., Hercules, CA). Western blot images were quantified using ImageJ software.

### NLRP3 immunofluorescence

NLRP3 distribution in cytosol was assessed by immunofluorescence techniques using antibodies against NLRP3 and DAPI as a marker of the nuclei.

### Nuclear morphology

For nuclear deformation cell quantification, nuclei were stained with DAPI. After staining, abnormal nuclei were counted in randomly selected fields (minimum one hundred cells per case) and expressed as percentages of total cells counted. Counting of cells with nuclear deformation was performed by three independent observers.

### Proliferation assay

@Fibroblasts were seeded in 12‐well plates. One hundred thousand fibroblasts were cultured with or without the MCC950 at two different concentrations (0.6 and 1.2 µM) for 24, 48, and 120 h. To measure proliferation rate, fibroblasts was seeded and after treatment, cells were harvested and quantified by using a TC10™ Automated Cell Counter (Bio‐Rad). Cell viability was assessed by trypan blue exclusion. Proliferation rate was calculated by total cell number. Results are expressed as mean ± SD of two independent assays.

### Animals

Animal studies were performed in accordance with European Union guidelines (2010/63/EU) and the corresponding Spanish regulations for the use of laboratory animals in chronic experiments (RD 53/2013 on the care of experimental animals). All experiments were approved by the local institutional animal care committee. For all experiments, only male mice were used. Mutant mice deficient in Zmpste24 metalloproteinase and Lmna^G609G/G609G^ have been described previously (Osorio *et al*, 2012). All groups had *ad libitum* access to their prescribed diet and water throughout the whole study. Body weight was monitored weekly. Animal rooms were maintained at 20–22°C with 30–70% relative humidity. As positive control, we used a gain of function NLRP3 mutant associated with neonatal onset multisystem inflammatory disease in mice (Bonar *et al*, 2012).

For all experiments with NLRP3 inhibitors, Zmpste24^+/+^ (wild type) and Zmpste24^−/−^ were maintained on a regular 12 h light/dark cycle at 20–22°C. Treatments were started at 1 month of age after randomization into three groups (wild‐type vehicle, Zmpste24^−/−^ vehicle, and Zmpste24^−/−^MCC950). These groups correspond to the following treatment: (i) standard diet with i.p vehicle (saline) treatment (vehicle groups) from Teklad Global 14% Protein Rodent Maintenance Diet, Harlan Laboratories (carbohydrate:protein:fat ratio of 48:14:4 percent of kcal) and (ii) standard diet with MCC950 treatment (MCC950 group). MCC950 was administered 20 mg/kg daily by i.p. route. All groups had *ad libitum* access to their prescribed diet and water throughout the study. Individuals were monitored daily and weighed monthly but were otherwise left undisturbed until they died. Survival was assessed using male mice, and all animals were dead by the time of this report. Kaplan–Meier survival curves were constructed using known birth and death dates, and differences between groups were evaluated using the log‐rank test. A separate group of male mice were sacrificed at age 4 months to study (Western blots).

### Statistical analysis

Data in the figures are shown as mean ± SD. Data between different groups were analyzed statistically by using ANOVA on ranks with Sigma plot and Sigma Stat statistical software (SPSS for Windows, 19, 2010, SPSS Inc. Chicago, IL, USA). Normality was assessed with the Kolmogorov–Smirnov test. In case of unequal variances, Welch's correction was applied. For cell‐culture studies, Student's *t*‐test was used for data analyses. A value of *P* < 0.05 was considered significant. In animal studies, the experiments were done after encoding the name of the groups.

## Author contributions

Conceptualization: MDC, EA‐G, JMN‐P, and GM. Investigation: AG‐D, RM, BC‐V, JN‐V, DL‐C, and CW. Formal analysis: AG‐D, RM, BC‐V, JN‐V, DL‐C, and CW. Writing—original draft: MDC, EA‐G, and GM. Supervision: MDC and EA‐G. Resources: MDC and GM. Funding acquisition: MDC.

## Conflict of interest

The authors declare that they have no conflict of interest.

**For more information**

https://www.progeriaresearch.org/

## Supporting information



Appendix

Expanded View Figures PDF

Source Data for Expanded View

Source Data for Figure 1

Source Data for Figure 2

## Data Availability

This study includes no data deposited in external repositories.
