# Supplementary material for: Inhibition of the NLRP3 inflammasome improves lifespan in animal murine model of Hutchinson–Gilford Progeria
Source: EMBO Mol Med. 2021 Aug 27;13(10):e14012. doi: 10.15252/emmm.202114012 (PMC8495449; doi:10.15252/emmm.202114012)
Supplement: Supplementary file 1 — Appendix [file EMMM-13-e14012-s002.pdf]

Appendix

1. Appendix Figure S1. NLRP3-inflammasome complex protein expression with more representative animals.
2. Appendix Figure S2. Densitometry of Figure 1 R2.
3. Appendix Figure S3. Densitometry of Figure 2 R2.
4. Appendix Figure S4. Effect of the MCC950 treatment in skin fibroblasts from HGPS patient 2.

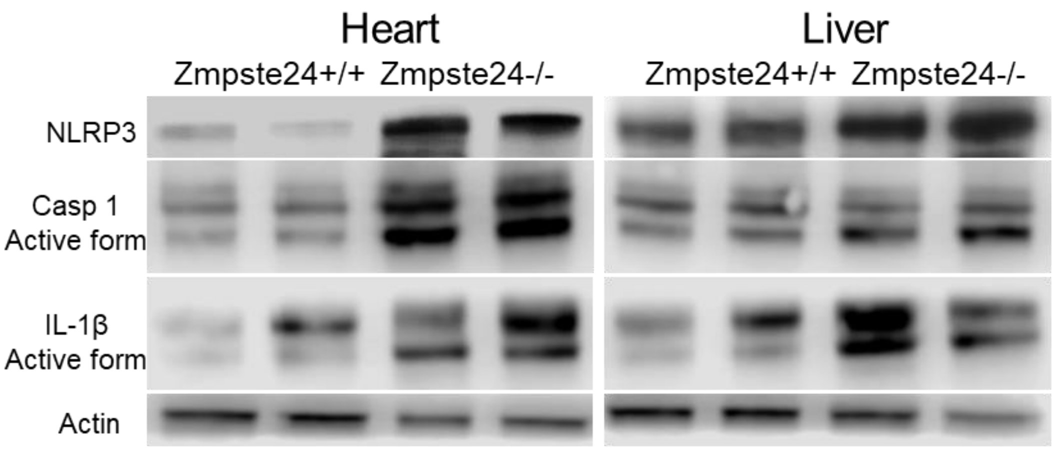

Appendix Figure S1. NLRP3-inflammasome complex protein expression with more representative animals. Western blot analysis with representative blot including NLRP3, caspase 1, IL-1β and actin levels in heart and liver tissues from wild-type and Zmpste24$^{-/-}$ mice.

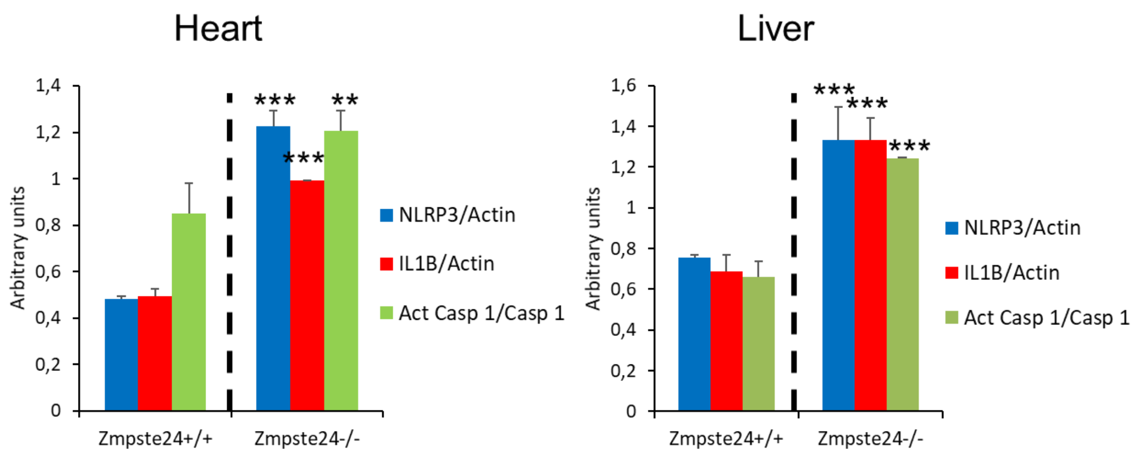

Appendix Figure S2. Densitometry of Figure 1 R2. Densitometric analysis is shown as means ± SD, n = 4 mice per group (2 in Figure 1 R2 and 2 in this figure). ***P < 0.001, **P < 0.005, *P < 0.05 wild-type *vs* Zmpste24$^{-/-}$ mice.

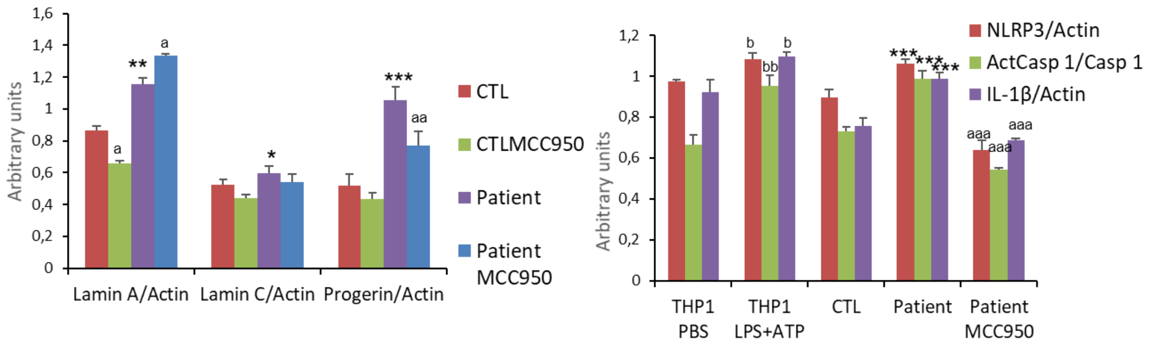

Appendix Figure S3. Densitometry of Figure 2 R2. Densitometric analysis is shown as means ± SD. Data represent 3 different experiments from the same patient. ***P < 0.001, **P < 0.005, *P < 0.05 control cells *vs* patient cells; ^{aaa}P < 0.001; ^{aa}P < 0.01; ^{a}P < 0.01 no treatment *vs* treatment; ^{bb}P < 0.005; ^{b}P < 0.05 THP cells no treatment *vs* treatment.

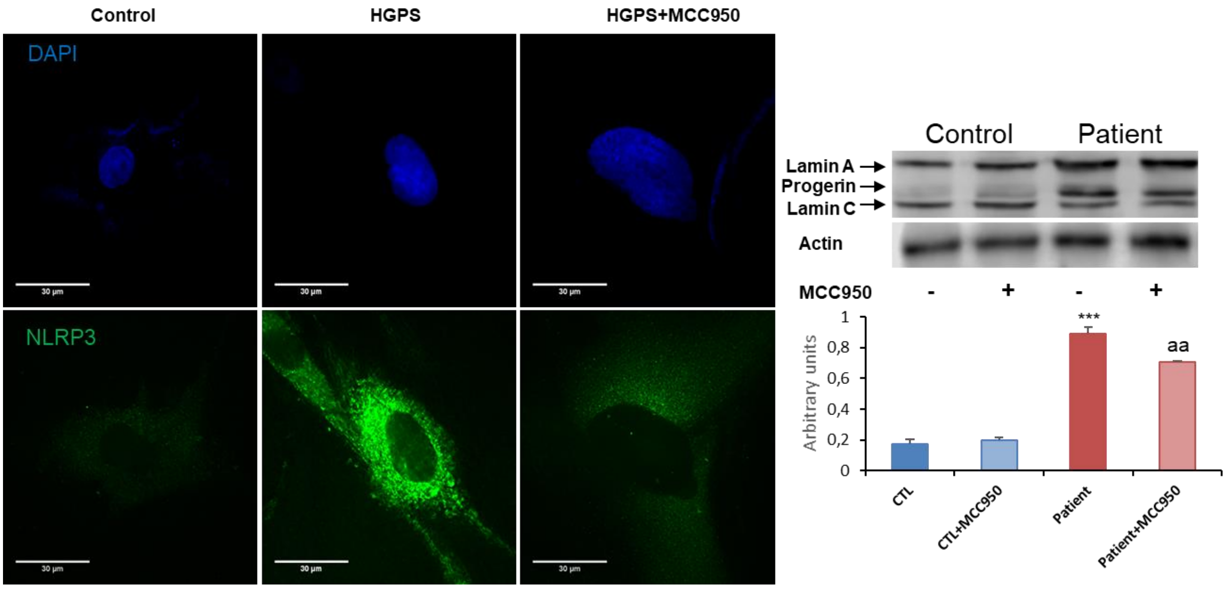

Appendix Figure S4. Effect of the MCC950 treatment in skin fibroblasts from HGPS patient 2. Data represent 3 different experiments from the same patient. ***P < 0.001, **P < 0.005, *P < 0.05 control cells *vs* patient cells; ^{aaa}P < 0.001; ^{aa}P < 0.01; ^{a}P < 0.01 no treatment *vs* treatment.
