# Supplementary material for: Inhibition of the NLRP3 inflammasome improves lifespan in animal murine model of Hutchinson–Gilford Progeria
Source: EMBO Mol Med. 2021 Aug 27;13(10):e14012. doi: 10.15252/emmm.202114012 (PMC8495449; doi:10.15252/emmm.202114012)
Supplement: Supplementary file 2 — Expanded View Figures PDF [file EMMM-13-e14012-s005.pdf]

# Expanded View Figures

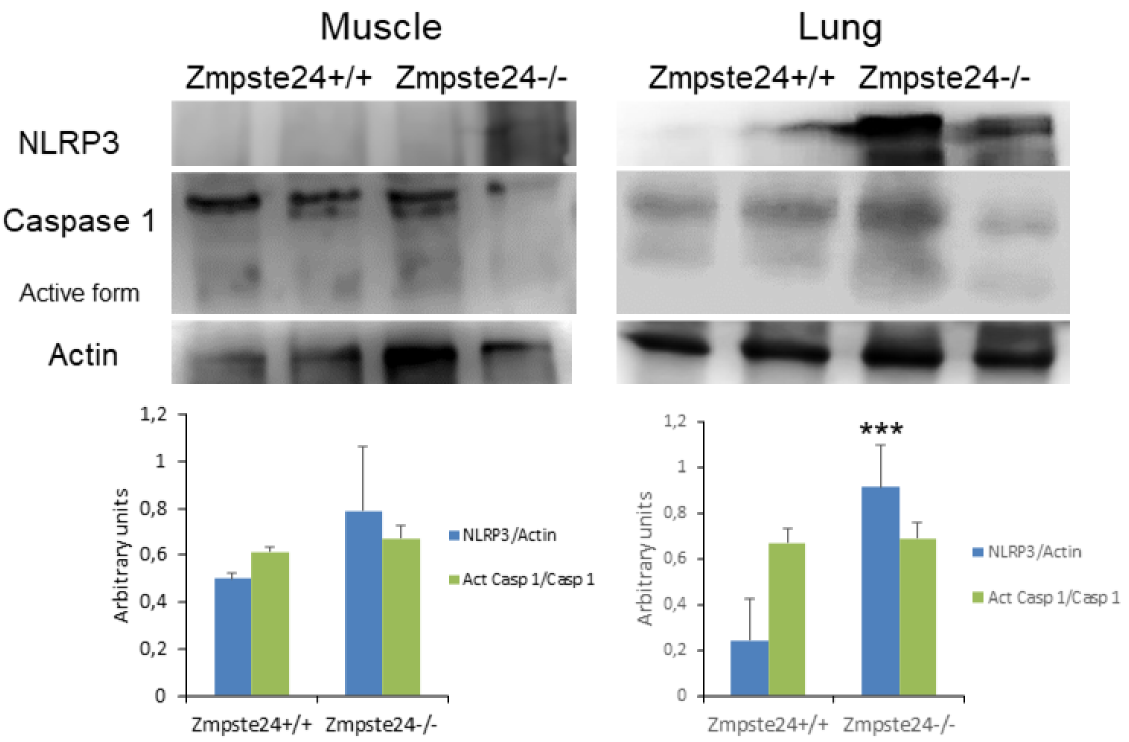


**Figure EV1. NLRP3 inflammasomes expression in lung and muscle from progeroid animals.**

Western blot analysis with representative blot including NLRP3, caspase 1, and actin levels in lung and muscle tissues from wild-type and Zmpste24$^{-/-}$ mice. Densitometric analysis is shown as means ± SD, $n$ = 5 mice per group. Data are shown as means ± SD. ***$P$ < 0.001, wild-type vs Zmpste24$^{-/-}$ mice. One-way ANOVA test was used for statistical analysis.

Source data are available online for this figure.

 © 2021 The Authors

 © 2021 The Authors

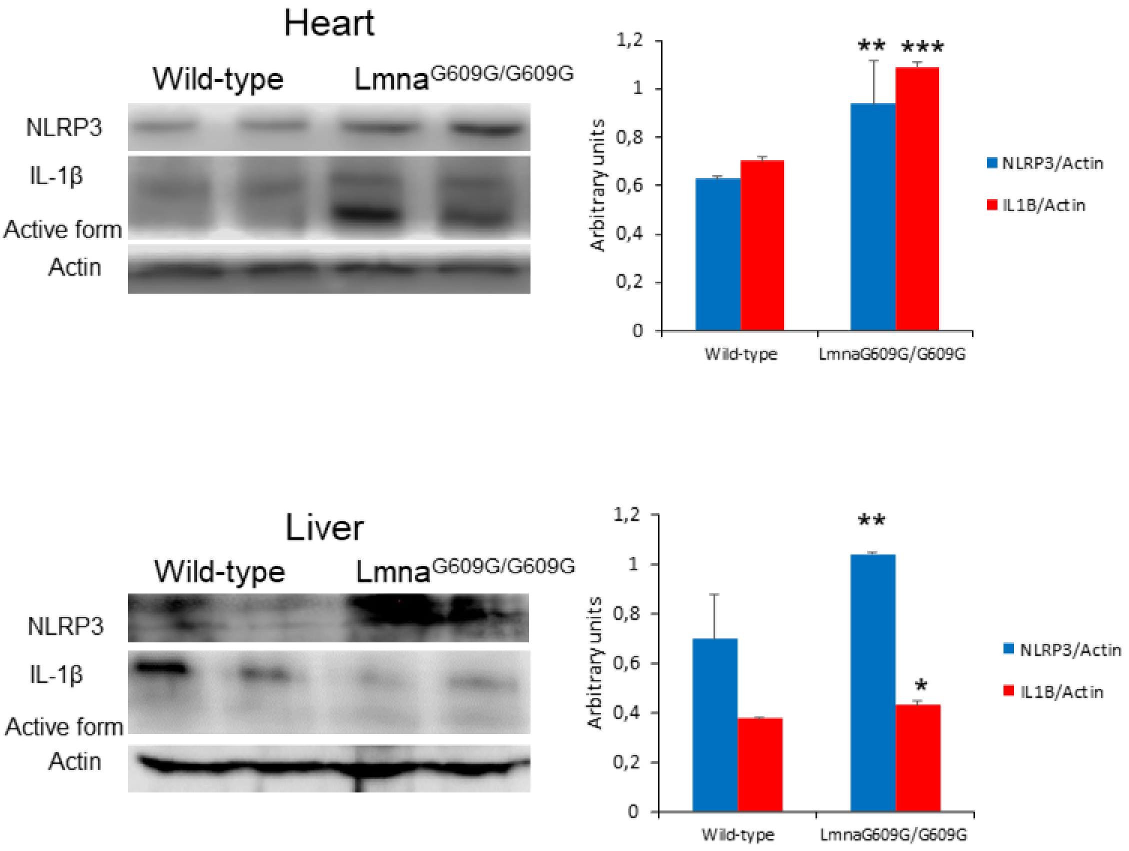


**Figure EV2. NLRP3 inflammasomes expression in heart and liver from Lmna$^{G609G/G609G}$ mice.**

Western blot analysis with representative blot including NLRP3, IL-1β, and actin levels in cardiac and liver tissues from wild-type and Lmna$^{G609G/G609G}$ mice. Densitometric analysis is shown as means ± SD, $n = 4$ mice per group. Data are shown as means ± SD. ***$P < 0.001$, **$P < 0.005$, *$P < 0.05$ wild-type vs Lmna$^{G609G/G609G}$ mice. One-way ANOVA test was used for statistical analysis.

Source data are available online for this figure.
