# Supplementary figures and images for: Inhibition of the NLRP3 inflammasome improves lifespan in animal murine model of Hutchinson–Gilford Progeria
Source: EMBO Mol Med. 2021 Aug 27;13(10):e14012. doi: 10.15252/emmm.202114012 (PMC8495449; doi:10.15252/emmm.202114012)

Figure EV3A Heart

NLRP3

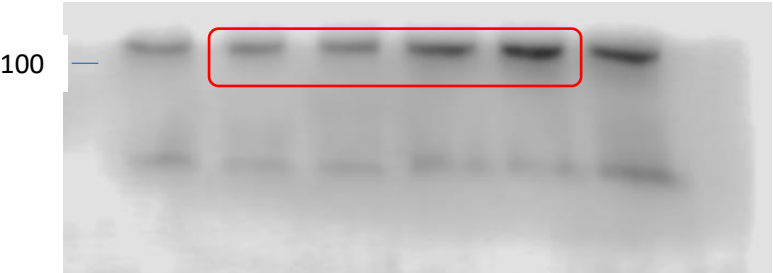

IL-1beta

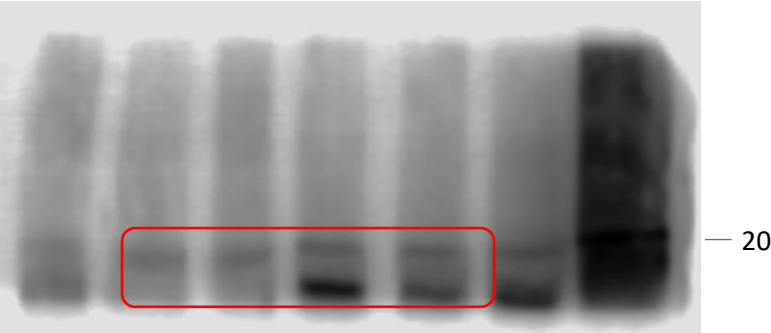

Actin

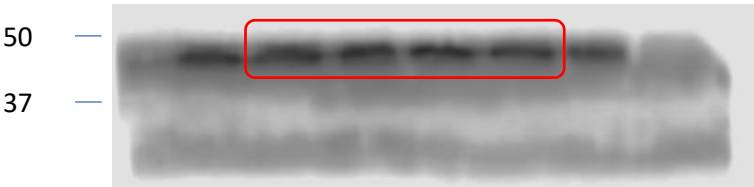

Figure EV3A Liver

NLRP3

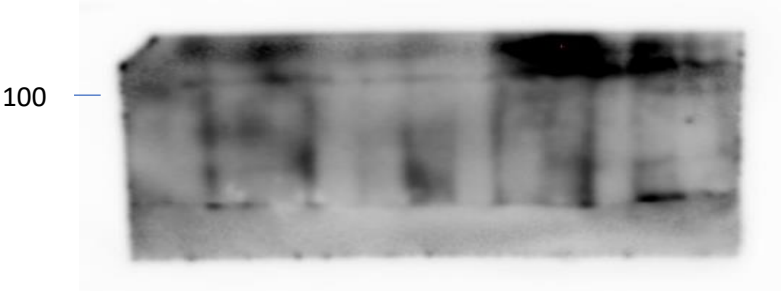

IL-1beta

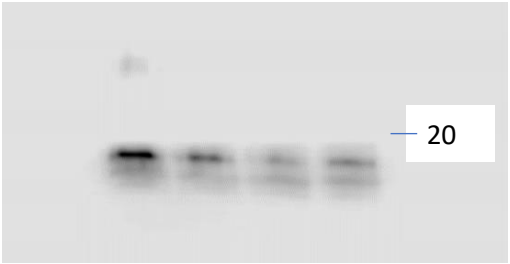

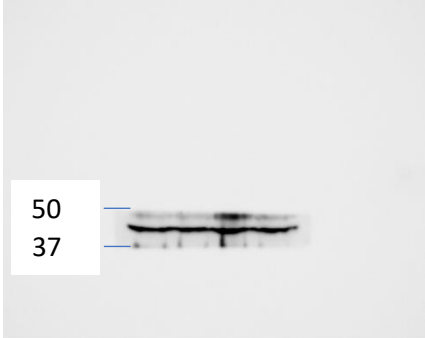

Supplement: Supplementary file 3 — Source Data for Expanded View [file EMMM-13-e14012-s006.zip › EMM-2021-14012-V4-Figure_EV2_Source_Data-sd.pdf]

Figure EV1 Muscle

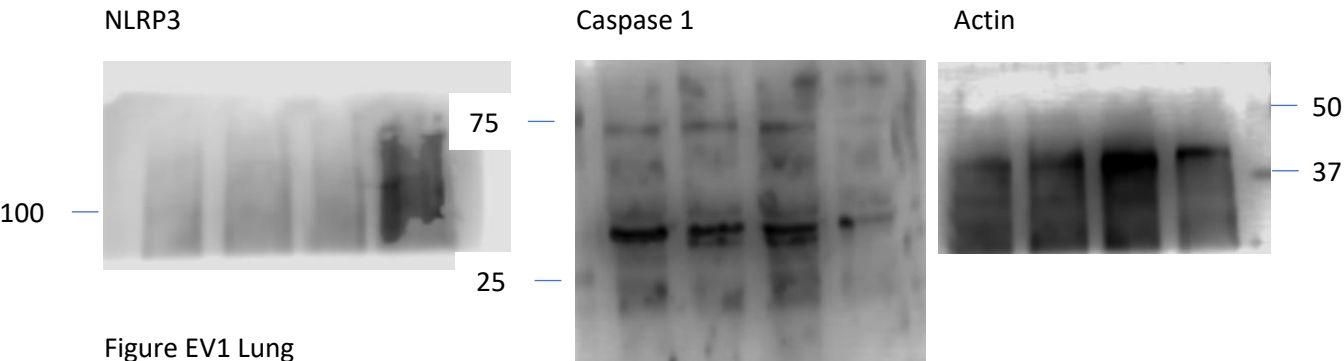

Figure EV1 Lung

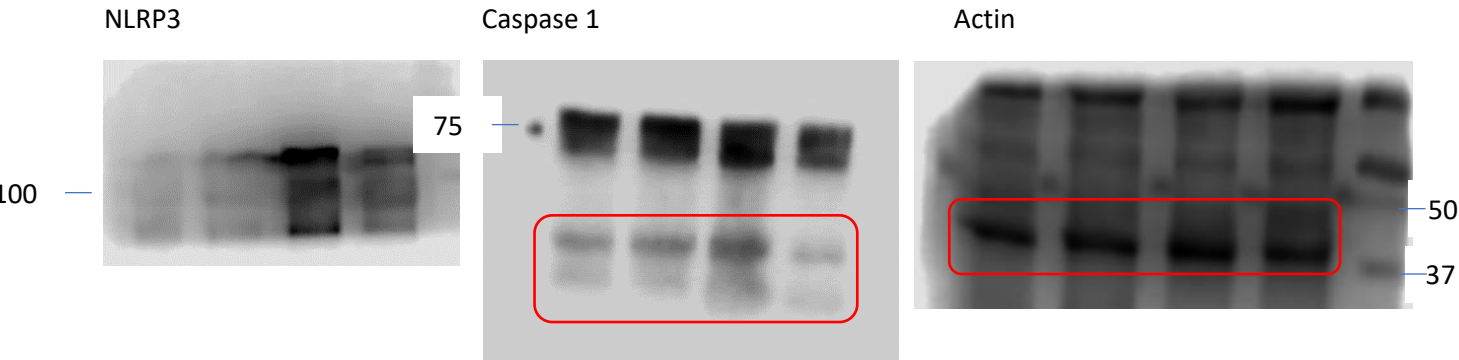

Supplement: Supplementary file 3 — Source Data for Expanded View [file EMMM-13-e14012-s006.zip › EMM-2021-14012-V4-Figure_EV1_Source_Data-sd.pdf]

Figure 1

Figure 1C. NLRP3

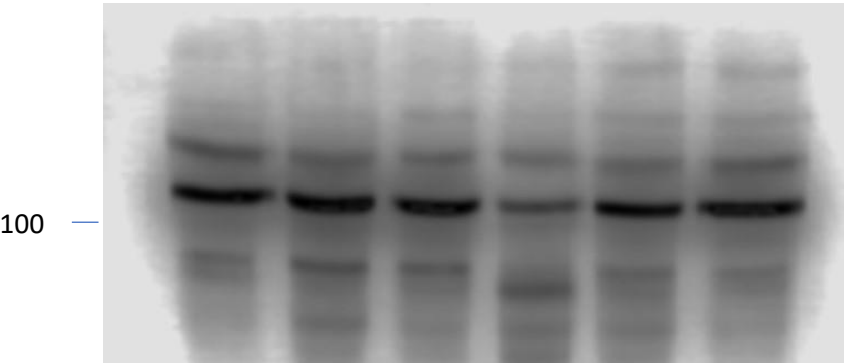

Figure 1C. Caspase 1

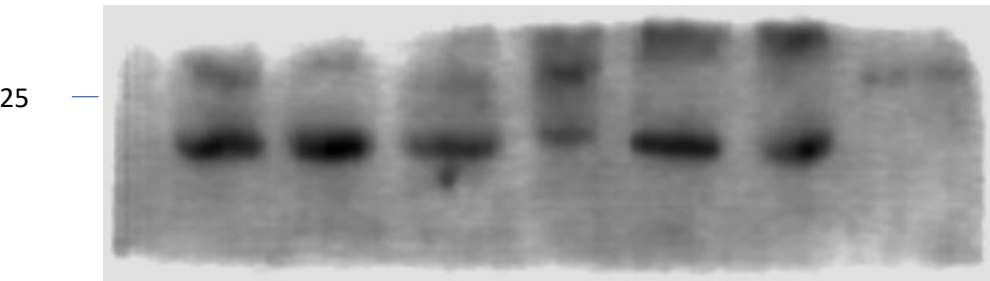

Figure 1C. Actin

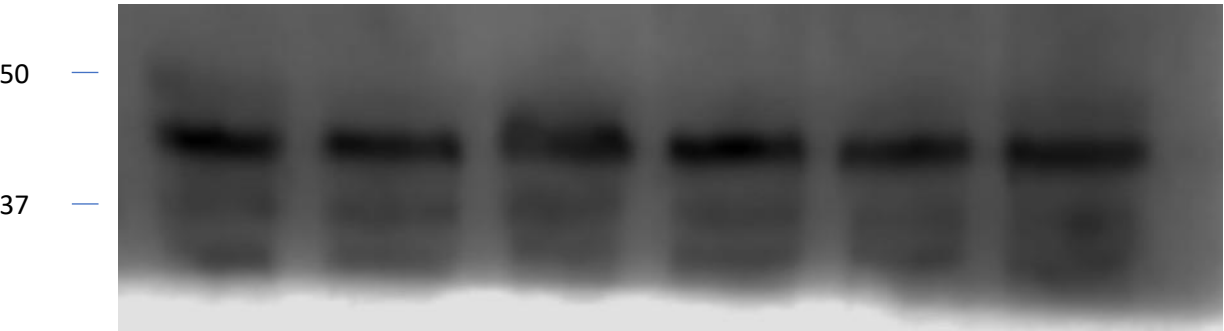

Supplement: Supplementary file 4 — Source Data for Figure 1 [file EMMM-13-e14012-s001.zip › EMM-2021-14012-V4-Figure_1C_Source_Data-sd.pdf]

Figure 2B

Lamin A/C

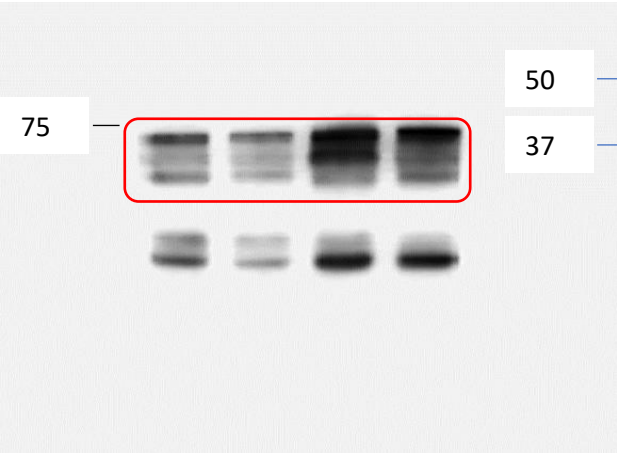

Actin

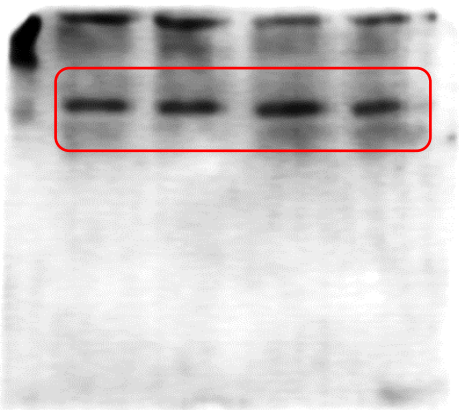

NLRP3

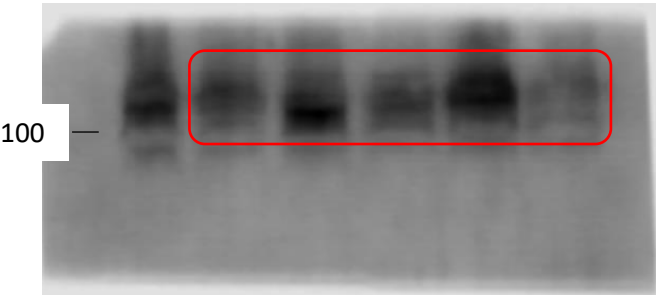

Caspase 1

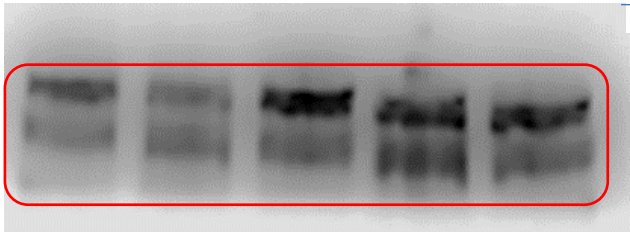

IL-1beta

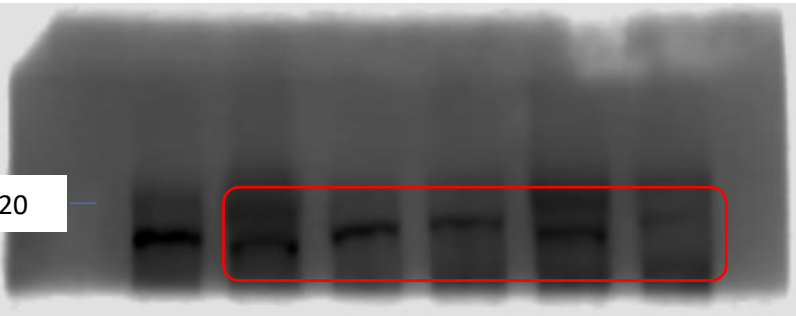

Actin

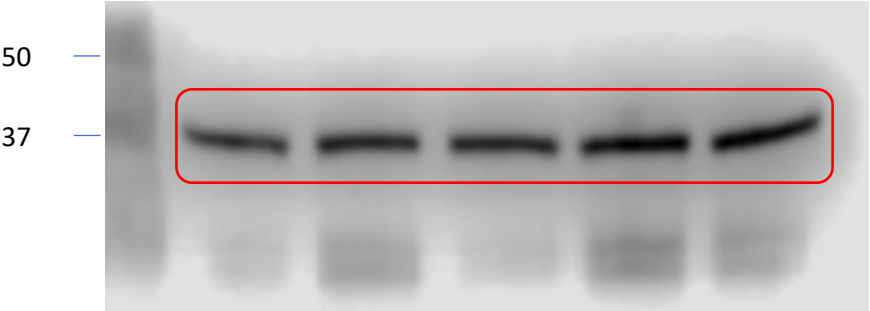

Supplement: Supplementary file 5 — Source Data for Figure 2 [file EMMM-13-e14012-s003.pdf]
