# Supplementary material for: Inhibition of the NLRP3 inflammasome improves lifespan in animal murine model of Hutchinson–Gilford Progeria
Source: EMBO Mol Med. 2021 Aug 27;13(10):e14012. doi: 10.15252/emmm.202114012 (PMC8495449; doi:10.15252/emmm.202114012)

FIGURE 1

Figure 1A. NLRP3

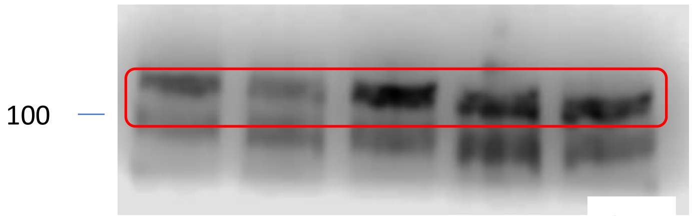

Figure 1A. Caspase 1

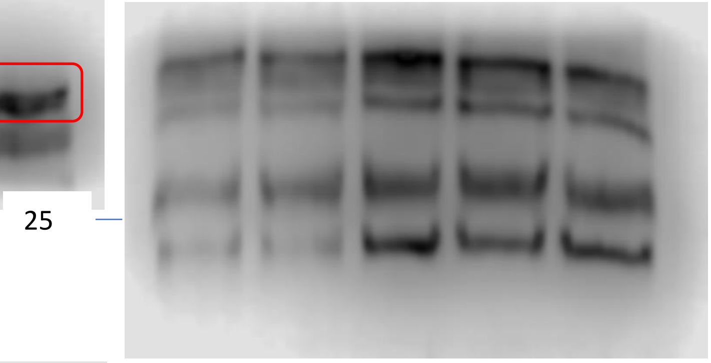

Figure 1A. Actin

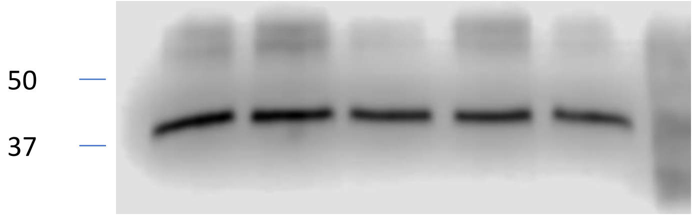

Figure 1A. Lamin A/C Patient 1

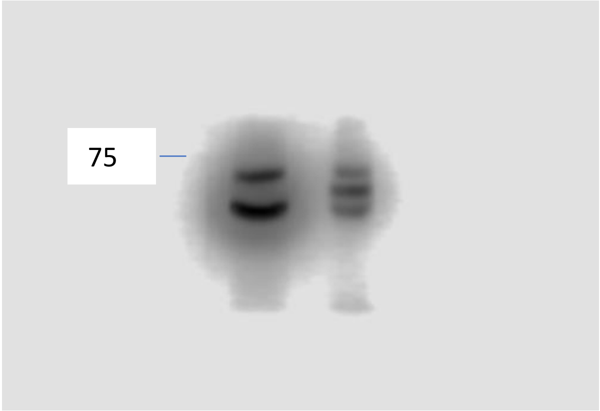

Figure 1A. Lamin A/C Patient 2

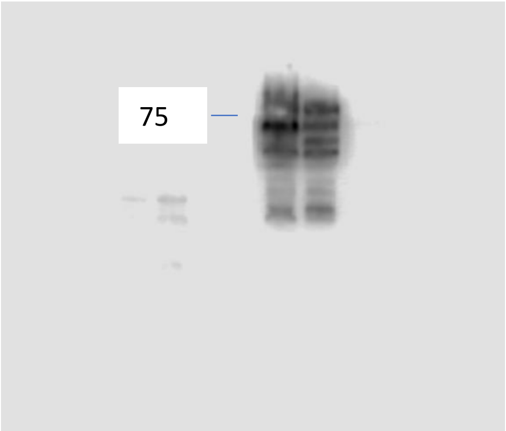

Figure 1A. Actin Patient 1

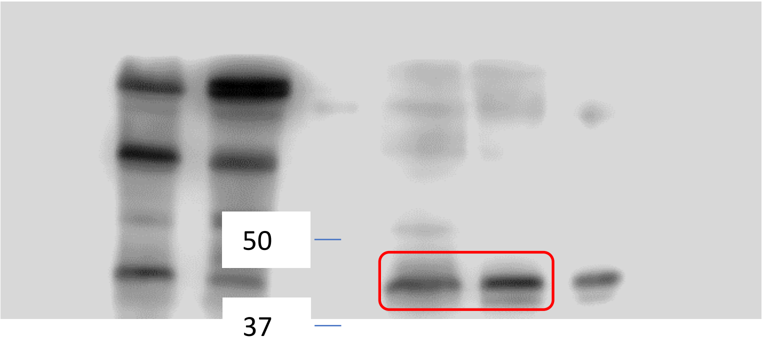

Figure 1A. Actin Patient 2

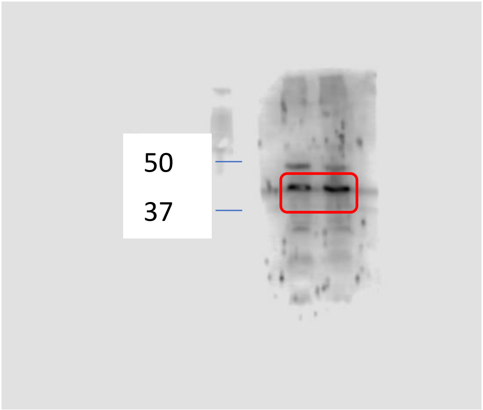

Supplement: Supplementary file 4 — Source Data for Figure 1 [file EMMM-13-e14012-s001.zip › EMM-2021-14012-V4-Figure_1A_Source_Data-sd.pdf]
