# Supplementary material for: Inhibition of the NLRP3 inflammasome improves lifespan in animal murine model of Hutchinson–Gilford Progeria
Source: EMBO Mol Med. 2021 Aug 27;13(10):e14012. doi: 10.15252/emmm.202114012 (PMC8495449; doi:10.15252/emmm.202114012)

Figure 1

Figure 1E. NLRP3 Heart

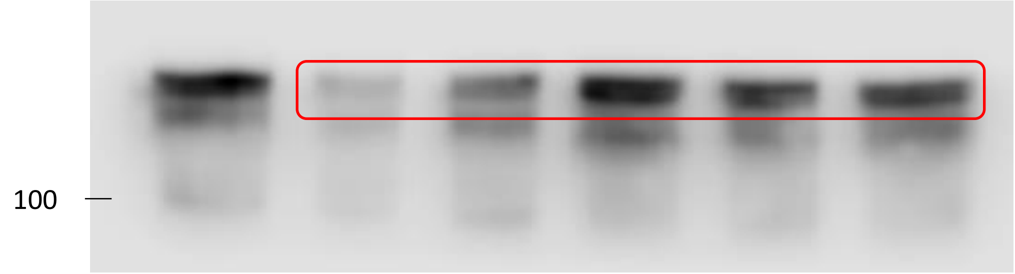

Figure 1E. Caspase 1 Heart

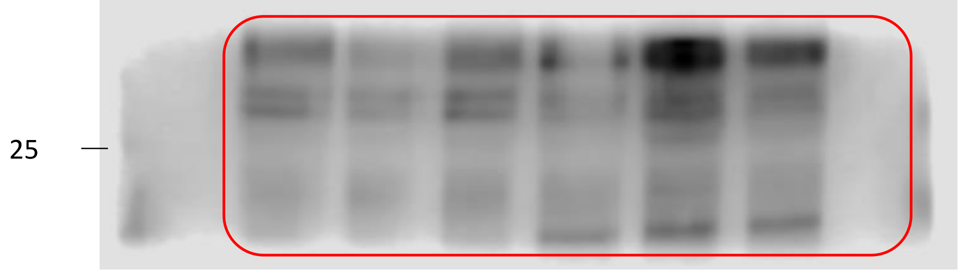

Figure 1E. IL-1beta Heart

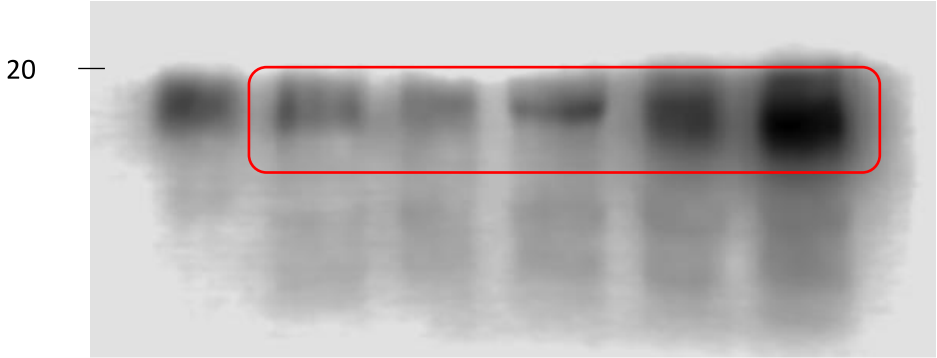

Figure 1E. Actin Heart

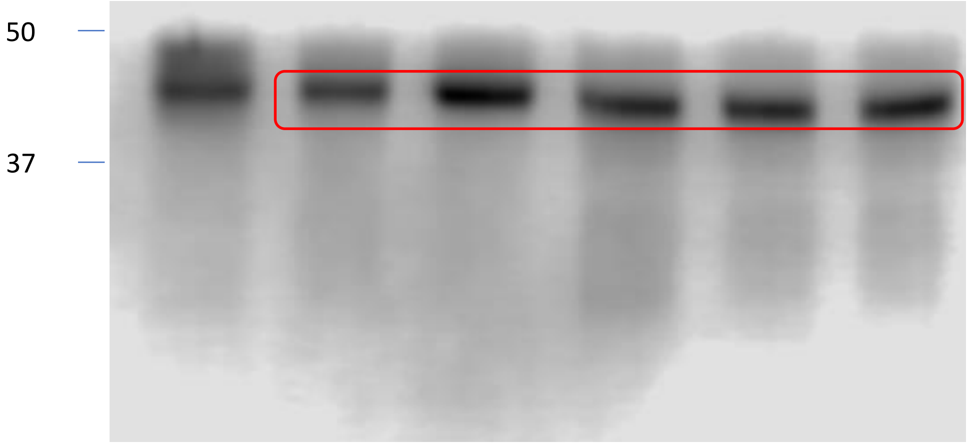

Figure 1E. NLRP3 Liver

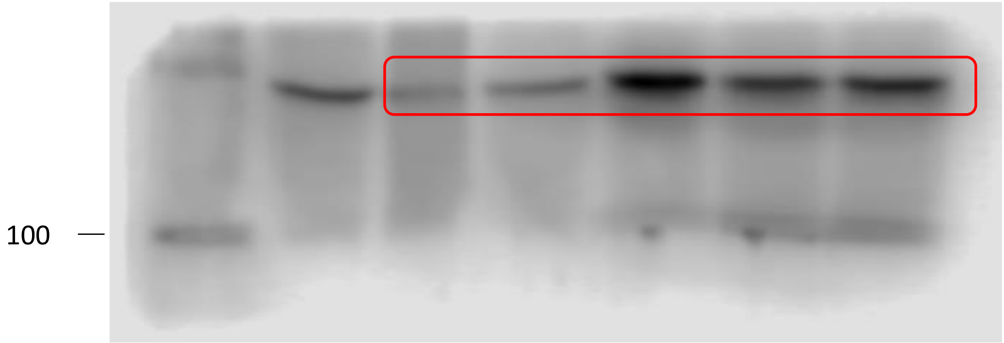

Figure 1E. Caspase 1 Liver

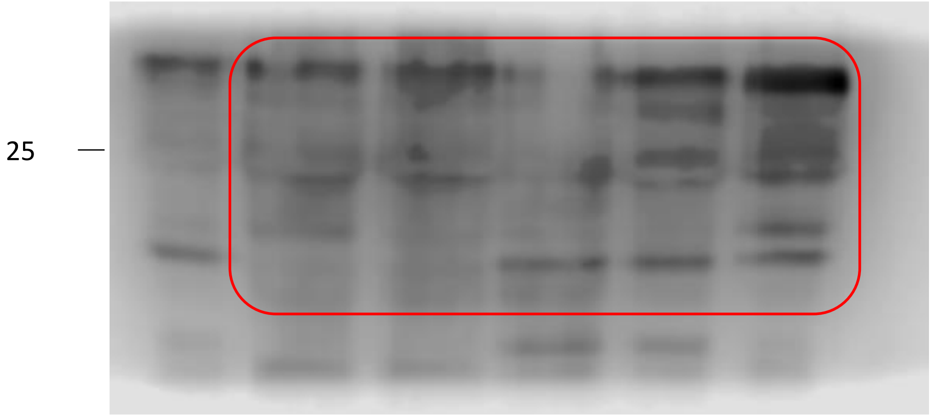

Figure 1E. IL-1beta Liver

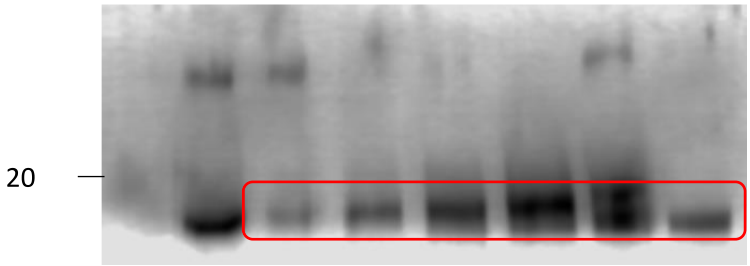

Figure 1E. Actin Liver

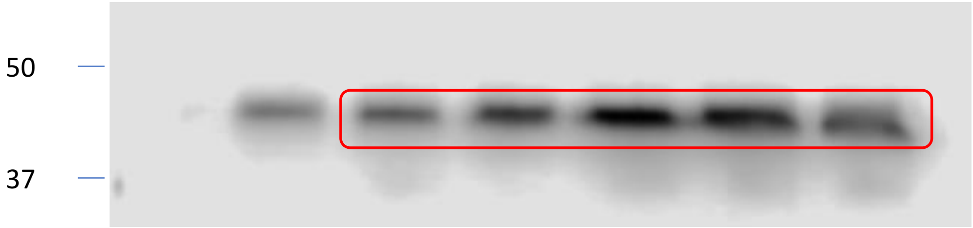

Supplement: Supplementary file 4 — Source Data for Figure 1 [file EMMM-13-e14012-s001.zip › EMM-2021-14012-V4-Figure_1E_Source_Data-sd.pdf]
