# Supplementary material for: Inhibition of the NLRP3 inflammasome improves lifespan in animal murine model of Hutchinson–Gilford Progeria
Source: EMBO Mol Med. 2021 Aug 27;13(10):e14012. doi: 10.15252/emmm.202114012 (PMC8495449; doi:10.15252/emmm.202114012)
Supplement: Supplementary file 6 [file EMMM-13-e14012-s004.pdf]

EMBO Molecular Medicine

Inhibition of the NLRP3 inflammasome improves lifespan in animal murine model of Hutchinson-Gilford Progeria

Alvaro González-Dominguez, Raul Montañez, Beatriz Castejón-Vega, Jessica Nunez-Vasco, Debora Lendines-Cordero, Chun Wang, Gabriel Mbalaviele, Jose Navarro-Pando, Elisabet Alcocer-Gómez, and Mario Cordero
DOI: 10.15252/emmm.202114012

Corresponding author: Mario Cordero (mario.cordero@inibica.es)

Review Timeline:

Submission Date:	22nd Jan 21
Editorial Decision:	10th Feb 21
Revision Received:	4th May 21
Editorial Decision:	18th May 21
Revision Received:	28th Jun 21
Editorial Decision:	22nd Jul 21
Revision Received:	9th Aug 21
Accepted:	10th Aug 21

Editor: Zeljko Durdevic

Transaction Report:

(Note: With the exception of the correction of typographical or spelling errors that could be a source of ambiguity, letters and reports are not edited. Depending on transfer agreements, referee reports obtained elsewhere may or may not be included in this compilation. Referee reports are anonymous unless the Referee chooses to sign their reports.)

10th Feb 2021

Dear Dr. Cordero,

Thank you for the re-submission of your manuscript to EMBO Molecular Medicine. We have now received feedback from the three reviewers who agreed to evaluate your manuscript. As you will see from the reports below, while the referee #2 is overall supporting the publication of the manuscript, referees #1 and #3 acknowledge the interest and improvements of the study but also raise serious concerns that should be addressed in a major revision. Particular attention should be given to improving the technical quality of the molecular analyses and to including appropriate controls to each experiment.

Addressing the reviewers' concerns in full will be necessary for further considering the manuscript in our journal, and acceptance of the manuscript will entail a second round of review. EMBO Molecular Medicine encourages a single round of revision only and therefore, acceptance or rejection of the manuscript will depend on the completeness of your responses included in the next, final version of the manuscript. For this reason, and to save you from any frustrations in the end, I would strongly advise against returning an incomplete revision.

We realize that the current situation is exceptional on the account of the COVID-19/SARS-CoV-2 pandemic. Therefore, please let us know if you need more than three months to revise the manuscript.

I look forward to receiving your revised manuscript.

Yours sincerely,

Zeljko Durdevic
Editor
EMBO Molecular Medicine

***** Reviewer's comments *****

Referee #1 (Comments on Novelty/Model System for Author):

See my comments below: molecular analyses lack controls to convincingly demonstrate inflammasome activation in progeria patients and mice.

Referee #1 (Remarks for Author):

In their revised manuscript, the authors have performed a number of additional experiments and analyses that overall improved the study. The main point of the study consists of an extension in the lifespan of a progeria mouse model upon treatment with the MCC950 Nlrp3 inhibitor. This indicates that Nlrp3 inflammasome activity contributes to progeria pathogenesis, but the technical quality of the molecular analyses corroborating this finding remain too poor to convince the reader of this concept. The authors should demonstrate Nlrp3 inflammasome activity in human and mouse progeria samples in a convincing manner to allow publication of this study. Below are my specific comments.

Major comments:

1. In response to my earlier question the authors have now provided a positive control for human caspase-1 processing in Fig 1A by adding a lysate from Thp1 cells stimulated with LPS + ATP. However, this positive control has no value when the corresponding negative controls (not stimulated, LPS alone, ATP alone) are not shown. According to the material and methods the authors performed a mock PBS control but they don't show it on the western blot. This negative control is needed to see whether the casp1 band at 20kDa is really the active p20 subunit, as this band should be absent in the negative control and present in the positive control.

2. The authors did not include controls for their murine Western blots as I had suggested. I can understand that the authors do not have experience themselves with inflammasome activation experiments in mouse macrophages and have no direct access to corresponding KO mice, but it shouldn't be that difficult to find a collaborator that provides control WB samples of mouse macrophages stimulated or not with an Nlrp3 agonist. This collaborator could also provide samples from Nlrp3KO or Casp1KO mice as controls for the authors' Western blots. As these blots look now they are not convincing. For instance, the blot in Fig 1S would suggest that WT naive mice display spontaneous Casp1 activation in muscle, more than Zmpste24-/- mice do... The authors need controls to make their murine active Casp1 and IL1b bands convincing. In order to support the presence of active Nlrp3 in Zmpste24-/- mice the authors could also blot samples of these mice together with samples from MCC950-treated Zmpste24-/- mice. If the bands they show are really the active forms of Casp1/IL1b they should be diminished in the samples from MCC950-treated Zmpste24-/- mice.

3. Despite my previous suggestion, the authors did not add controls to the Western blot in Fig 2B (the above Thp1 lysates could be used), and they didn't probe the membrane for Casp1 activation.

4. As suggested also by reviewer 1, when the authors perform densitometric analyses of Western blots of 5 different mice and only show 2 mice, they could provide a supplementary figure showing all the blots used for the analyses.

Minor comments:

1. If it is really true that you had the manuscript revised by a native English speaker then please never rely on this person anymore. The paper is still full of typo's and grammatical errors.

2. Fig S2: Please justify why you investigate only Nlrp1, Nlrp10 and Nlrp4, while there are many other inflammasome sensors such as Nlrc4, Nlrp6, Aim2, Pyrin, ... Why did you measure Nlrp4, since this has not been described to be a real inflammasome sensor?

3. On page 9 you write that Zmpste24-/- display an increment in Nlrp3 expression in heart and liver but not in lungs and muscle. However, in Fig 1S you show more Nlrp3 expression in lungs of Zmpste24-/- mice when compared to WT mice.

Referee #2 (Remarks for Author):

n the revised manuscript, the authors answered the critiques and added additional experiments and analysis as needed to respond to the issue raised. These include relatively better quality western blots, and corroborate the NLRP3-inflammasome activation with laminG609G/G609G mouse model. Overall, I find the response to my comments satisfactory, and the added experimental data convincing. The paper has significantly improved upon revision.

Referee #3 (Remarks for Author):

Alcocer-Gomez et al. show activation of NLRP3 and Caspase 1, as well as cytokines Il-1b and IL-18 in HGPS patients-derived cells and in some tissues of progeria mouse models. Inhibition of inflammasome with MCC950 improves proliferation and nuclear abnormalities in progeria cells in vitro and extends healthspan and lifespan of progeria mice. The in vivo result is novel and important, as new therapies for HGPS are needed that improve patients' health. Although the manuscript has improved since last submission, some weaknesses are identified that should be addressed.

Recommendations:

• Increased levels of NLRP3 and Caspase 1 are shown in liver and heart of Zmpste24-/- mice but not in muscle and lung. In LmnaG609G/G609G mice, NLRP3 and Il-1b are upregulated in heart. The question is why the authors did not monitor the other markers or other tissues in LmnaG609G/G609G mice. It should be stated whether they looked and there was not an effect, or if they didn't look. It would be important to know if the result in not the same as in Zmpste24-/-. It will be interesting either way because it might reveal some differences between models.
• Graph with densitometry in Figure 1E difficult to read.
• Forgot to discuss Figure 1F in the text.
• In Figure 2A, cell growth is graphed as percentage of control cells. Not clear how control cells increase over time. It is a bit confusing. Total number of cells over time or population doublings would be a clearer way to show the data. In addition, the cell growth data from the second patient (Fig S4) could be combined with the data from patient one (Fig 2A). Also combining all the data with the 2 concentrations would be best to determine whether there is a statistically significant dose-dependent effect on cell growth. By the way, the statistics of the experiment are not included in figure.
• Graph with densitometry in Figure 2B difficult to read.

We would like to thank you and the reviewers for the time spent to review our paper. We have now produced a revised version of the manuscript in which we have addressed the reviewers' comments and implemented the majority of their suggestions.

We appreciate the suggestions by the reviewer 1 about the inclusion of the appropriate controls. However, as you can see in the bibliography in similar journals to EMBO Mol Med, this is usually not necessary, especially in aging studies: PMID: 33113366, PMID: 32964663, PMID: 32666684, PMID: 24093676, PMID: 33097533, PMID: 33231615. Despite this, we have included the most significant controls such as THP1 treated with LPS+ATP or NOMID mice with NLRP3 gain-of-function mutation. We have repeated all Western blot analyses, which include the appropriate controls, and think that the main conclusions of our work are still valuable.

Below is our point-by-point reply to each reviewer.

Referee #1 (Remarks for Author):

In their revised manuscript, the authors have performed a number of additional experiments and analyses that overall improved the study. The main point of the study consists of an extension in the lifespan of a progeria mouse model upon treatment with the MCC950 Nlrp3 inhibitor. This indicates that Nlrp3 inflammasome activity contributes to progeria pathogenesis, but the technical quality of the molecular analyses corroborating this finding remain too poor to convince the reader of this concept. The authors should demonstrate Nlrp3 inflammasome activity in human and mouse progeria samples in a convincing manner to allow publication of this study. Below are my specific comments.

Major comments:

1. In response to my earlier question the authors have now provided a positive control for human caspase-1 processing in Fig 1A by adding a lysate from Thp1 cells stimulated with LPS + ATP. However, this positive control has no value when the corresponding negative controls (not stimulated, LPS alone, ATP alone) are not shown. According to the material and methods the authors performed a mock PBS control but they don't show it on the western blot. This negative control is needed to see whether the casp1 band at 20kDa is really the active p20 subunit, as this band should be absent in the negative control and present in the positive control.

We thank to the reviewer for this comment. The mock PBS control was included in the experiment, but was not included in the figure because it was placed in the first column of the blots. We have now included it based on the reviewer's advice.

2. The authors did not include controls for their murine Western blots as I had suggested. I can understand that the authors do not have experience themselves with inflammasome activation experiments in mouse macrophages and have no direct access to corresponding KO mice, but it shouldn't be that difficult to find a collaborator that provides control WB samples of mouse macrophages stimulated or not with an Nlrp3 agonist. This collaborator could also

provide samples from Nlrp3KO or Casp1KO mice as controls for the authors' Western blots. As these blots look now they are not convincing. For instance, the blot in Fig 1S would suggest that WT naive mice display spontaneous Casp1 activation in muscle, more than Zmpste24-/- mice do... The authors need controls to make their murine active Casp1 and IL1b bands convincing. In order to support the presence of active Nlrp3 in Zmpste24-/- mice the authors could also blot samples of these mice together with samples from MCC950-treated Zmpste24-/- mice. If the bands they show are really the active forms of Casp1/IL1b they should be diminished in the samples from MCC950-treated Zmpste24-/- mice.

Again, we appreciate the reviewer's suggestions. Certainly, the inclusion of a positive control to compare the NLRP3-inflammasome complex activation help to understand this process. However, as you can see in the bibliography, this is usually not necessary, especially in aging studies: PMID: 33113366, PMID: 32964663, PMID: 32666684, PMID: 24093676, PMID: 33097533, PMID: 33231615. We think that our data suggest that the NLRP3 inflammasome is implicated in HGPS. We recognize that more studies will need to be performed in the future, but we think that the main conclusions of our work are valid. However, based on the reviewer's recommendation, we have now included a positive control (THP1 cells stimulated with LPS+ATP) to figure 1A. Regarding a positive control proposed in mice, we have now included data obtained with neonatal onset multisystem inflammatory disease (NOMID) in mice, which express NLRP3 with a gain-of- function mutation.

Furthermore, blots of muscle samples have been repeated and included.

3. Despite my previous suggestion, the authors did not add controls to the Western blot in Fig 2B (the above Thp1 lysates could be used), and they didn't probe the membrane for Casp1 activation.

We have now included a positive control.

4. As suggested also by reviewer 1, when the authors perform densitometric analyses of Western blots of 5 different mice and only show 2 mice, they could provide a supplementary figure showing all the blots used for the analyses.

We have improved the quality of the blots in this new version based on previous recommendations. We have now performed densitometric analyses using the blots from the second and this new version; positive controls are now included. The other blots are in the supplementary data as suggested by the reviewer.

Minor comments:

1. If it is really true that you had the manuscript revised by a native English speaker then please never rely on this person anymore. The paper is still full of typo's and grammatical errors.

The paper has been revised by a native English speaker who is also co-author of this manuscript for his contributions to the studies of NOMID mice

2. Fig S2: Please justify why you investigate only Nlrp1, Nlrp10 and Nlrp4, while there are many other inflammasome sensors such as Nlrc4, Nlrp6, Aim2, Pyrin, ... Why did you measure Nlrp4, since this has not been described to be a real inflammasome sensor?

NLRP4 has been shown to assemble a functional inflammasome (PMID: 22928810; PMID: 23192004).

3. On page 9 you write that Zmpste24-/- display an increment in Nlrp3 expression in heart and liver but not in lungs and muscle. However, in Fig 1S you show more Nlrp3 expression in lungs of Zmpste24-/- mice when compared to WT mice.

We agree with this comment and apologize for the oversight. This has been added to the revised manuscript.

Referee #2 (Remarks for Author):

In the revised manuscript, the authors answered the critiques and added additional experiments and analysis as needed to respond to the issue raised. These include relatively better quality western blots, and corroborate the NLRP3-inflammasome activation with laminG609G/G609G mouse model. Overall, I find the response to my comments satisfactory, and the added experimental data convincing. The paper has significantly improved upon revision.

We would like to thank to the reviewer because his/her comments have helped to improve our manuscript.

Referee #3 (Remarks for Author):

Alcocer-Gomez et al. show activation of NLRP3 and Caspase 1, as well as cytokines Il-1b and IL-18 in HGPS patients-derived cells and in some tissues of progeria mouse models. Inhibition of inflammasome with MCC950 improves proliferation and nuclear abnormalities in progeria cells in vitro and extends healthspan and lifespan of progeria mice. The in vivo result is novel and important, as new therapies for HGPS are needed that improve patients' health. Although the manuscript has improved since last submission, some weaknesses are identified that should be addressed.

Recommendations:

• Increased levels of NLRP3 and Caspase 1 are shown in liver and heart of Zmpste24-/- mice but not in muscle and lung. In LmnaG609G/G609G mice, NLRP3 and Il-1b are upregulated in heart. The question is why the authors did not monitor the other markers or other tissues in LmnaG609G/G609G mice. It should be stated whether they looked and there was not an effect, or if they didn't look. It would be important to know if the result in not the same as in Zmpste24-/-. It will be interesting either way because it might reveal some differences between models.

We thank the reviewer for this comment. Unfortunately, it was impossible to get several tissues from this mouse model. However, we managed to get hearts and livers from these

animals. The corresponding data have now been included in supplementary figure. We will check the effects of NLRP3 inhibitors in these mice in future studies.

• Graph with densitometry in Figure 1E difficult to read.

Densitometry has been improved and moved to supplementary figure.

• Forgot to discuss Figure 1F in the text.

Thank you very much for this observation. We have now included in the results section (Page 9).

• In Figure 2A, cell growth is graphed as percentage of control cells. Not clear how control cells increase over time. It is a bit confusing. Total number of cells over time or population doublings would be a clearer way to show the data. In addition, the cell growth data from the second patient (Fig S4) could be combined with the data from patient one (Fig 2A). Also combining all the data with the 2 concentrations would be best to determine whether there is a statistically significant dose-dependent effect on cell growth. By the way, the statistics of the experiment are not included in figure.

We have done the experiment, quantified cells by TC10™ Automated Cell Counter (Bio-Rad), and combined data as suggested by the reviewer (Figure 2 R2).

• Graph with densitometry in Figure 2B difficult to read.

Similar to the Figure 1F, densitometry has been improved and moved to supplementary figure.

18th May 2021

Dear Dr. Cordero,

Thank you for the submission of your revised manuscript to EMBO Molecular Medicine. We have now heard back from the two referees who we asked to re-evaluate your manuscript. As you will see from the reports below, the referees are acknowledging improvements of your revised manuscript but also raise serious concerns that should be addressed in an additional and final round of major revision. Please address all the points raised by the referees and implement all suggested adjustments. Particular attention should be given to improving technical quality of the molecular analyses specifically western blotting.

Further consideration of a revision that addresses reviewers' concerns in full will entail an additional round of review. Acceptance or rejection of the manuscript will depend on the completeness of your responses included in the next, final version of the manuscript. For this reason, and to save you from any frustrations in the end, I would strongly advise against returning an incomplete revision. I look forward to receiving your revised manuscript.

I also note that this second round of major revision will require a substantial amount of additional work. I am unsure whether you will be able or willing to address those and return a revised manuscript within the 3 months deadline. Therefore, I would also understand your decision if you chose to rather seek rapid publication elsewhere at this stage. Should you find that the requested revisions are not feasible within the constraints outlined here and choose, therefore, to submit your paper elsewhere, we would welcome a message to this effect.

Yours sincerely,

Zeljko Durdevic

Zeljko Durdevic
Editor
EMBO Molecular Medicine

***** Reviewer's comments *****

Referee #1 (Comments on Novelty/Model System for Author):

The quality of the inflammasome activation analyses by western blotting remains very low. I believe the overall conclusion of the study, but based on my own experience with these analyses and based on the issues raised by the novel Fig 2B (see comment 2 below) I still doubt whether the bands claimed to be Nlrp3, active casp1 and IL-1bb p17 really represent these proteins.

Referee #1 (Remarks for Author):

In their revised manuscript, the authors included additional samples and repeated some of the Western blots. While the paper improved, the technical quality of these molecular analyses remains poor to convince the reader of increased inflammasome activation. In addition, adding controls in MCC950 experiments in Fig 2B casted new doubts on this concept. Below are my specific comments on how the manuscript should be improved.

Major comments:

1. In response to one of my earlier questions the authors have now provided blots for IL-1b in Fig 1E and 2B. However, these blots in Fig 1E and 2B only show the active form of IL-1b. The authors should provide the full blots to be able to appreciate also the pro-form of IL-1b. This will discriminate effects on priming versus inflammasome activation, and it will increase the confidence that the band the authors are showing is really the active p17 band of IL-1b.

2. In response to my previous suggestion, the authors now added Thp1 controls to the Western blot in Fig 2B. They added Thp1 lysates untreated or treated with MCC950. While the latter is an Nlrp3 inflammasome inhibitor, it did not diminish caspase-1 activation or the maturation of IL-1b in Thp1 cells according to the blots provided by the authors. Can the authors explain this? In addition, can the authors discuss the mode of action of MCC950 in the patient cells? MCC950 is an inhibitor that targets the ATPase domain of Nlrp3 and thereby inhibits Nlrp3 activation (see https://www.nature.com/articles/s41589-019-0277-7). This activity of MCC950 does not affect stability of the Nlrp3 protein. On the contrary, MCC950 protects both mouse Nlrp3 and human NLRP3 from protease-mediated degradation (see Fig S2b in https://www.nature.com/articles/s41589-019-0277-7). Yet, the authors show in Fig 2B that in their patient cells NLRP3 was entirely absent upon MCC950 treatment. Does MCC950 adopt a different mode of action in patient cells? In addition, MCC950 treatment of patient cells according to the authors' Western blots in Fig 2B diminished casp1 activation to the levels of the healthy subject, yet

the maturation of IL-1b was absent in the patient cells but still present in the healthy subject cells. Can the authors explain these confusing Western blotting results?

Minor comments:

1. The authors still did not explain why they investigated Nlrp1, Nlrp10 and Nlrp4 expression, while there are more accepted inflammasome sensors such as Nlrc4, Nlrp6, Aim2, Pyrin, ... Especially their focus on Nlrp4 remains strange since Nlrp4 is not an inflammasome sensor. In their response, the authors claim that Nlrp4 is an inflammasome sensor by referring to PMID 22928810 but in fact this paper shows that Nlrp4 does not bind ASC, thereby reducing the odds that it can assemble an inflammasome. The authors also refer to PMID 23192004, which in fact deals with NLRC4 that they name by its ancient name NLRP4. NLRP4 (=Pypaf4) is different from NLRC4 (=Ipaf). The increased expression of Nlrp1, Nlrp10 and Nlrp4 (which also would not automatically mean activation) is confusing for the Nlrp3 focus of the study. How do the authors explain the MCC950 in vivo effect in their mouse model if also these other inflammasomes are activated? In my opinion, the focus and overall credibility of this study for the inflammasome research field would increase when the authors would omit the Nlrp1, Nlrp10 and Nlrp4 observations from the manuscript.

Referee #3 (Remarks for Author):

The manuscript has been improved since the last submission.
However, a few caveats still remain:
In figure 1F, it is not specified how many samples of heart and liver were used in the q-RT-PCR analysis of NLRP3 and Caspase 1.
In figure 2B, the WB+densitometry analysis is performed in the cells from one patient. Why not in both? Differences of phenotype?
How many lines of HGPS and control fibroblasts used in Fig 2C-E? Probably only one?
Statistical analysis needed in graphs in Fig 2A and also in Kaplan Meier curve and body weight curve (Fig 2F and 2G).

We would like to thank you and the reviewers for the time spent to review our paper. We have now produced a revised version of the manuscript in which we have addressed the reviewers' comments and implemented the majority of their suggestions.

We have included in this letter an experiment to explain the effect of MCC950 on NLRP3 protein levels, which has also been reported by others (PMID: 30381407 (Fig. 6E), PMID: 28167322 (Fig. 2A and F), PMID: 30277570 (Fig. 5A), PMID: 32223388 (Fig. 2E), PMID: 32507974 (Fig.2B), PMID: 29495433 (Fig. 5A), PMID: 30716395 (Fig.2A), PMID: 33676524 (Fig. 3C)).

Below is our point-by-point reply to each reviewer.

Referee #1 (Remarks for Author):

In their revised manuscript, the authors included additional samples and repeated some of the Western blots. While the paper improved, the technical quality of these molecular analyses remains poor to convince the reader of increased inflammasome activation. In addition, adding controls in MCC950 experiments in Fig 2B casted new doubts on this concept. Below are my specific comments on how the manuscript should be improved.

Major comments:

1. In response to one of my earlier questions the authors have now provided blots for IL-1b in Fig 1E and 2B. However, these blots in Fig 1E and 2B only show the active form of IL-1b. The authors should provide the full blots to be able to appreciate also the pro-form of IL-1b. This will discriminate effects on priming versus inflammasome activation, and it will increase the confidence that the band the authors are showing is really the active p17 band of IL-1b.

We understand this comment. However, we would like to mention that the mature form of IL-1b (p17) is not easy to detect in tissue lysates because it is secreted. To help with the recognition of the p17-related band, we have included a positive control from NOMID mice as suggested by the reviewer.

2. In response to my previous suggestion, the authors now added Thp1 controls to the Western blot in Fig 2B. They added Thp1 lysates untreated or treated with MCC950. While the latter is an Nlrp3 inflammasome inhibitor, it did not diminish caspase-1 activation or the maturation of IL-1b in Thp1 cells according to the blots provided by the authors. Can the authors explain this?

Our apology for the mistake. The THP1 cell lysates that we originally included as controls, actually corresponded to THP1+PBS and THP1+LPS+ATP (positive control). THP1 cells treated with MCC950 were not included by error.

In addition, can the authors discuss the mode of action of MCC950 in the patient cells? MCC950 is an inhibitor that targets the ATPase domain of Nlrp3 and thereby inhibits Nlrp3 activation (see https://www.nature.com/articles/s41589-019-0277-7). This activity of

MCC950 does not affect stability of the Nlrp3 protein. On the contrary, MCC950 protects both mouse Nlrp3 and human NLRP3 from protease-mediated degradation (see Fig S2b in https://www.nature.com/articles/s41589-019-0277-7). Yet, the authors show in Fig 2B that in their patient cells NLRP3 was entirely absent upon MCC950 treatment. Does MCC950 adopt a different mode of action in patient cells? In addition, MCC950 treatment of patient cells according to the authors' Western blots in Fig 2B diminished casp1 activation to the levels of the healthy subject, yet the maturation of IL-1b was absent in the patient cells but still present in the healthy subject cells. Can the authors explain these confusing Western blotting results?

We thank the reviewer for the comments and discussion. Although the effects of MCC950 have been extensively reports on potential off-targets of this inhibitor are emerging as described in this paper (PMID: 34003636). Other papers have also described with the effect of MCC950 in decreasing NLRP3 protein levels in different diseases and models: PMID: 30381407 (Fig. 6E), PMID: 28167322 (Fig. 2A and F), PMID: 30277570 (Fig. 5A), PMID: 32223388 (Fig. 2E), PMID: 32507974 (Fig.2B), PMID: 29495433 (Fig. 5A), PMID: 30716395 (Fig.2A), PMID: 33676524 (Fig. 3C). We think that MCC950 affects the priming of NLRP3; this effect is lost once the NLRP3 inflammasome complex is assembled. In other words, MCC950 exerts inhibitory effects of NLRP3 expression when it is used before or at the same time of priming with LPS for example; this effect of MCC950 is lost when it is added to cells after LPS+ATP treatment. We also think that in pathological conditions such as progeria or in models used in the aforementioned paper, MCC950 treatment prevents the formation of new inflammasome complexes while those assembled before MCC950 exposure are degraded through autophagy or other proteolytic mechanisms. In fact, high levels of basal autophagy have been showed in HGPS models (PMID: 18443001, PMID: 20886757).

To strengthen our findings, we have done the following experiment:

CTL: Control cells,

L+A: LPS+ATP-treated cells (10µg/ml LPS for 4 hours+5mM ATP for 30 minutes)

M+L+A: MCC950 (1.2µM) for 15 minutes before L+A, then LPS 10µg/ml for 4 hours then then ATP (5mM) for 30 minutes.

L+A+M: LPS (10µg/ml) for 4 hours followed by ATP (5mM) for 30 minutes then MCC950 (1.2µM) for 15 minutes.

Figure for referees removed

These results clearly shows the effect of MCC950 depends on the time of its addition to cells. We have not included these data to the manuscript because they are outside the scope of this work.

Minor comments:

1. The authors still did not explain why they investigated Nlrp1, Nlrp10 and Nlrp4 expression, while there are more accepted inflammasome sensors such as Nlrc4, Nlrp6, Aim2, Pyrin, ... Especially their focus on Nlrp4 remains strange since Nlrp4 is not an inflammasome sensor. In their response, the authors claim that Nlrp4 is an inflammasome sensor by referring to PMID 22928810 but in fact this paper shows that Nlrp4 does not bind ASC, thereby reducing the odds that it can assemble an inflammasome. The authors also refer to PMID 23192004, which in fact deals with NLRC4 that they name by its ancient name NLRP4. NLRP4 (=Pypaf4) is different from NLRC4 (=Ipaf). The increased expression of Nlrp1, Nlrp10 and Nlrp4 (which also would not automatically mean activation) is confusing for the Nlrp3 focus of the study. How do the authors explain the MCC950 in vivo effect in their mouse model if also these other inflammasomes are activated? In my opinion, the focus and overall credibility of this study for the inflammasome research field would increase when the authors would omit the Nlrp1, Nlrp10 and Nlrp4 observations from the manuscript.

We have removed data related to NLRP1, NLRP10, and NLRP4 as recommended by the reviewer.

Referee #3 (Remarks for Author):

The manuscript has been improved since the last submission.

However, a few caveats still remain:

In figure 1F, it is not specified how many samples of heart and liver were used in the q-RT-PCR analysis of NLRP3 and Caspase 1.

This information has been included accordingly

In figure 2B, the WB+densitometry analysis is performed in the cells from one patient. Why not in both? Differences of phenotype?

Because this is a brief report, we only included patient 1 as a representative data. However, we have included progerin levels of patient 2 in Appendix Figure S4.

How many lines of HGPS and control fibroblasts used in Fig 2C-E? Probably only one?

We have used two patients. The image shows a representative patient of both. However, we have included image and progerin levels of patient 2 in Appendix Figure S4.

Statistical analysis needed in graphs in Fig 2A and also in Kaplan Meier curve and body weight curve (Fig 2F and 2G).

All statistical analysis has been included accordingly.

2nd Revision - Editorial Decision 22nd Jul 2021

22nd Jul 2021

Dear Dr. Cordero,

Thank you for the submission of your revised manuscript to EMBO Molecular Medicine. I am pleased to inform you that we will be able to accept your manuscript pending the following final amendments:

1) Please address all the referee #1 suggestions.
2) In the main manuscript file, please do the following:
- Correct/answer the track changes suggested by our data editors by working from the attached document.
- Add up to 5 keywords.
- Remove font colour.
- Make sure that all special characters display well.
- Add contribution for all authors.
- Rename "Author Disclosure Statemen" to "Conflict of interest".
- In M&M, Include a statement that informed consent was obtained from all human subjects and that the experiments conformed to the WMA Declaration of Helsinki and to the principles set out in the Department of Health and Human Services Belmont Report.
- In M&M, statistical paragraph should reflect all information that you have filled in the Authors Checklist, especially regarding randomization, blinding, replication etc.
- Correct the reference citation in the text and reference list. In the text of the manuscript, a reference should be cited by author and year of publication. Include a space between a word and the opening parenthesis of the reference that follows. In the reference list, citations should be listed in alphabetical order. Where there are more than 10 authors on a paper, 10 will be listed, followed by "et al.". Please check "Author Guidelines" for more information.
https://www.embopress.org/page/journal/17574684/authorguide#referencesformat
- Add data availability statement. If no data are deposited in public repositories, please add the sentence: "This study includes no data deposited in external repositories". Please check "Author Guidelines" for more information.
https://www.embopress.org/page/journal/17574684/authorguide#availabilityofpublishedmaterial
3) Funding: Please make sure that information about all sources of funding are complete in both our submission system and in the manuscript.
4) Source data: Please upload 1 file per figure.
5) Synopsis: Please check your synopsis text and image, revise them if necessary and submit the final versions with your revised manuscript. Please be aware that in the proof stage minor corrections only are allowed (e.g., typos). Please submit synopsis text as a separate .doc file.
6) For more information: There is space at the end of each article to list relevant web links for further consultation by our readers. Could you identify some relevant ones and provide such information as well? Some examples are patient associations, relevant databases, OMIM/proteins/genes links, author's websites, etc...
7) As part of the EMBO Publications transparent editorial process initiative (see our Editorial at http://embomolmed.embopress.org/content/2/9/329), EMBO Molecular Medicine will publish online a Review Process File (RPF) to accompany accepted manuscripts. This file will be published in conjunction with your paper and will include the anonymous referee reports, your point-by-point response and all pertinent correspondence relating to the manuscript. Let us know whether you agree with the publication of the RPF and as here, if you want to remove or not any figures from it

prior to publication. Please note that the Authors checklist will be published at the end of the RPF.
8) Please provide a point-by-point letter INCLUDING my comments as well as the reviewer's reports and your detailed responses (as Word file).

I look forward to reading a new revised version of your manuscript as soon as possible.

Yours sincerely,

Zeljko Durdevic

Zeljko Durdevic
Editor
EMBO Molecular Medicine

***** Reviewer's comments *****

Referee #1 (Remarks for Author):

Dear Authors,

this manuscript might be accepted for publication after the following adjustments.

1. You did not respond to my previous request to show the pro-IL1b bands in Fig 1E and 2B. Please do so. I am not asking for new experiments or controls. Just show the higher molecular weight bands on the IL1b p17 blots so the reader can also see the pro-forms to appreciate the differential impacts on inflammasome priming and activation.

2. Page 6 line 3: fig 1D does not show IL1b and IL18 expression. It shows their secretion. Please correct and also describe the results a bit more careful: patient cells show more IL1b and IL18 secretion than control cells in naive conditions, but not upon inflammasome activation by uric acid or cholesterol crystals.

3. Page 6 middle: 'the NLRP3 inflammasome was also activated in the heart and liver in the LaminG609G/G609G mouse model (Figure EV2)'. This figure shows activation the heart but not in the liver, since there is no visible mature IL1b band in the liver lysates.

4. Fig 2C: please indicate on the pictures what you mean with 'abnormal nuclear morphology'. For non-specialists this is not clear from the DAPI stainings in this figure.

5. There is a Spanish comment DISCUTIR RESULTADO NUESTRO on page 9.

Referee #3 (Remarks for Author):

Okay to publish

Reviewer: 1

Referee #1 (Comments on Novelty/Model System for Author):

1.You did not respond to my previous request to show the pro-IL1b bands in Fig 1E and 2B.Please do so. I am not asking for new experiments or controls. Just show the higher molecularweight bands on the IL1b p17 blots so the reader can also see the pro-forms to appreciate thedifferential impacts on inflammasome priming and activation.

According to our previous letter, we have used an antibody which only recognizes the active form of IL1b p17 from Cell signaling (D3A3Z). So, we have not the pro-IL1b bands. However, If the reviewer and editor think that this is necessary, we can to repeat the blots using to antibodies.

2.Page 6 line 3: fig 1D does not show IL1b and IL18 expression. It shows their secretion.Please correct and also describe the results a bit more careful: patient cells show more IL1band IL18 secretion than control cells in naive conditions, but not upon inflammasomeactivation by uric acid or cholesterol crystals.

The text has been modified accordingly

3.Page 6 middle: 'the NLRP3 inflammasome was also activated in the heart and liver in the LaminG609G/G609G mouse model (Figure EV2)'. This figure shows activation the heart butnot in the liver, since there is no visible mature IL1b band in the liver lysates.

The text has been modified accordingly

4.Fig 2C: please indicate on the pictures what you mean with 'abnormal nuclear morphology'.For non-specialists this is not clear from the DAPI stainings in this figure.

The images from the Figure 2 has been modified accordingly

5.There is a Spanish comment DISCUTIR RESULTADO NUESTRO on page 9.

The text has been corrected accordingly

3rd Revision - Editorial Decision 10th Aug 2021

We are pleased to inform you that your manuscript is accepted for publication and is now being sent to our publisher to be included in the next available issue of EMBO Molecular Medicine.

EMBO PRESS

YOU MUST COMPLETE ALL CELLS WITH A PINK BACKGROUND 🡻

PLEASE NOTE THAT THIS CHECKLIST WILL BE PUBLISHED ALONGSIDE YOUR PAPER

Corresponding Author Name: Mario D. Cordero

Journal Submitted to: EMBO Molecular Medicine

Manuscript Number: EMM-2021-14012

Reporting Checklist For Life Sciences Articles (Rev. June 2017)

This checklist is used to ensure good reporting standards and to improve the reproducibility of published results. These guidelines are consistent with the Principles and Guidelines for Reporting Preclinical Research issued by the NIH in 2014. Please follow the journal's authorship guidelines in preparing your manuscript.

A- Figures

1. Data

The data shown in figures should satisfy the following conditions:

- the data were obtained and processed according to the field's best practice and are presented to reflect the results of the experiments in an accurate and unbiased manner.
- figure panels include only data points, measurements or observations that can be compared to each other in a scientifically meaningful way.
- graphs include clearly labeled error bars for independent experiments and sample sizes. Unless justified, error bars should not be shown for technical replicates.
- if n< 5, the individual data points from each experiment should be plotted and any statistical test employed should be justified
- Source Data should be included to report the data underlying graphs. Please follow the guidelines set out in the author ship guidelines on Data Presentation.

2. Captions

Each figure caption should contain the following information, for each panel where they are relevant:

- a specification of the experimental system investigated (eg cell line, species name).
- the assay(s) and method(s) used to carry out the reported observations and measurements
- an explicit mention of the biological and chemical entity(ies) that are being measured.
- an explicit mention of the biological and chemical entity(ies) that are altered/varied/perturbed in a controlled manner.

- the exact sample size (n) for each experimental group/condition, given as a number, not a range;
- a description of the sample collection allowing the reader to understand whether the samples represent technical or biological replicates (including how many animals, litters, cultures, etc.).
- a statement of how many times the experiment shown was independently replicated in the laboratory.
- definitions of statistical methods and measures:
  - common tests, such as t-test (please specify whether paired vs. unpaired), simple $\chi 2$ tests, Wilcoxon and Mann-Whitney tests, can be unambiguously identified by name only, but more complex techniques should be described in the methods section;
  - are tests one-sided or two-sided?
  - are there adjustments for multiple comparisons?
  - exact statistical test results, e.g., P values = x but not P values < x;
  - definition of 'center values' as median or average;
  - definition of error bars as s.d. or s.e.m.

Any descriptions too long for the figure legend should be included in the methods section and/or with the source data.

In the pink boxes below, please ensure that the answers to the following questions are reported in the manuscript itself. Every question should be answered. If the question is not relevant to your research, please write NA (non applicable). We encourage you to include a specific subsection in the methods section for statistics, reagents, animal models and human subjects.

USEFUL LINKS FOR COMPLETING THIS FORM

http://www.antibodypedia.com
http://1degreebio.org
http://www.equator-network.org/reporting-guidelines/improving-bioscience-research-repor
http://grants.nih.gov/grants/olaw/olaw.htm
http://www.mrc.ac.uk/Ourresearch/Ethicsresearchguidance/Useofanimals/index.htm
http://ClinicalTrials.gov
http://www.consort-statement.org
http://www.consort-statement.org/checklists/view/32-consort/66-title
http://www.equator-network.org/reporting-guidelines/reporting-recommendations-for-tumo
http://datadryad.org
http://figshare.com
http://www.ncbi.nlm.nih.gov/gap
http://www.ebi.ac.uk/ega
http://biomodels.net/
http://biomodels.net/miriam/
http://jjj.biochem.sun.ac.za
http://oba.od.nih.gov/biosecurity/biosecurity_documents.html
http://www.selectagents.gov/

B- Statistics and general methods

Please fill out these boxes 🡻 (Do not worry if you cannot see all your text once you press return)

1.a. How was the sample size chosen to ensure adequate power to detect a pre-specified effect size?	This was using Graphpad analysis and according to previous papers
1.b. For animal studies, include a statement about sample size estimate even if no statistical methods were used.	N/A
2. Describe inclusion/exclusion criteria if samples or animals were excluded from the analysis. Were the criteria pre-established?	N/A
3. Were any steps taken to minimize the effects of subjective bias when allocating animals/samples to treatment (e.g. randomization procedure)? If yes, please describe.	Yes, the experiments were done after encoding the names so that the experimenter did not know the groups
For animal studies, include a statement about randomization even if no randomization was used.	
4.a. Were any steps taken to minimize the effects of subjective bias during group allocation or/and when assessing results (e.g. blinding of the investigator)? If yes please describe.	As previously commented, several of the experiment were done with blinding of the investigator
4.b. For animal studies, include a statement about blinding even if no blinding was done	
5. For every figure, are statistical tests justified as appropriate?	Yes
Do the data meet the assumptions of the tests (e.g., normal distribution)? Describe any methods used to assess it.	Normality was assessed with the Kolmogorov–Smirnov test.
Is there an estimate of variation within each group of data?	Yes

Is the variance similar between the groups that are being statistically compared?	Yes, an in case of unequal variances, a Welch's correction was applied

C- Reagents

6. To show that antibodies were profiled for use in the system under study (assay and species), provide a citation, catalog number and/or clone number, supplementary information or reference to an antibody validation profile. e.g., Antibodypedia (see link list at top right), 1DegreeBio (see link list at top right).	Lamin A/C sc-7292, NLRP3 D4D8T, NLRP1 NBP1-54899, NLRP10 MAS-24260, IL-1BETA D3A3Z, NLRP4 PA5-21020, Caspase 1 sc-56036 and Actin sc-47778
7. Identify the source of cell lines and report if they were recently authenticated (e.g., by STR profiling) and tested for mycoplasma contamination.	All cell line used in this study were from Progeria Research Foundation Cell and Tissue Bank and were tested for mycoplasma contamination with negative results.

* for all hyperlinks, please see the table at the top right of the document

D- Animal Models

8. Report species, strain, gender, age of animals and genetic modification status where applicable. Please detail housing and husbandry conditions and the source of animals.	We used mutant mice deficient in Zmpste24 metalloproteinase and LmnaG609G/G609G with C57BL6 backgrounds provided by Dr. Carlos López-Otin´s group. All groups had ad libitum access to their prescribed diet and water throughout the whole study. Body weight was monitored weekly. Animal rooms were maintained at 20–22°C with 30–70% relative humidity.
9. For experiments involving live vertebrates, include a statement of compliance with ethical regulations and identify the committee(s) approving the experiments.	Animal studies were performed in accordance with European Union guidelines (2010/63/EU) and the corresponding Spanish regulations for the use of laboratory animals in chronic experiments (RD 53/2013 on the care of experimental animals). All experiments were approved by the local institutional animal care committee from the Universidad de Cádiz.
10. We recommend consulting the ARRIVE guidelines (see link list at top right) (PLoS Biol. 8(6), e1000412, 2010) to ensure that other relevant aspects of animal studies are adequately reported. See author guidelines, under 'Reporting Guidelines'. See also: NIH (see link list at top right) and MRC (see link list at top right) recommendations. Please confirm compliance.	Yes

E- Human Subjects

11. Identify the committee(s) approving the study protocol.	N/A
12. Include a statement confirming that informed consent was obtained from all subjects and that the experiments conformed to the principles set out in the WMA Declaration of Helsinki and the Department of Health and Human Services Belmont Report.	N/A
13. For publication of patient photos, include a statement confirming that consent to publish was obtained.	N/A
14. Report any restrictions on the availability (and/or on the use) of human data or samples.	N/A
15. Report the clinical trial registration number (at ClinicalTrials.gov or equivalent), where applicable.	N/A
16. For phase II and III randomized controlled trials, please refer to the CONSORT flow diagram (see link list at top right) and submit the CONSORT checklist (see link list at top right) with your submission. See author guidelines, under 'Reporting Guidelines'. Please confirm you have submitted this list.	N/A
17. For tumor marker prognostic studies, we recommend that you follow the REMARK reporting guidelines (see link list at top right). See author guidelines, under 'Reporting Guidelines'. Please confirm you have followed these guidelines.	N/A

F- Data Accessibility

18: Provide a "Data Availability" section at the end of the Materials & Methods, listing the accession codes for data generated in this study and deposited in a public database (e.g. RNA-Seq data: Gene Expression Omnibus GSE39462, Proteomics data: PRIDE PXD000208 etc.) Please refer to our author guidelines for 'Data Deposition'.<br><br>Data deposition in a public repository is mandatory for:<br>a. Protein, DNA and RNA sequences<br>b. Macromolecular structures<br>c. Crystallographic data for small molecules<br>d. Functional genomics data<br>e. Proteomics and molecular interactions	N/A
19. Deposition is strongly recommended for any datasets that are central and integral to the study; please consider the journal's data policy. If no structured public repository exists for a given data type, we encourage the provision of datasets in the manuscript as a Supplementary Document (see author guidelines under 'Expanded View' or in unstructured repositories such as Dryad (see link list at top right) or Figshare (see link list at top right).	TOTAL BLOTS WERE PROVIDED IN SOURCE DATA
20. Access to human clinical and genomic datasets should be provided with as few restrictions as possible while respecting ethical obligations to the patients and relevant medical and legal issues. If practically possible and compatible with the individual consent agreement used in the study, such data should be deposited in one of the major public access-controlled repositories such as dbGAP (see link list at top right) or EGA (see link list at top right).	N/A
21. Computational models that are central and integral to a study should be shared without restrictions and provided in a machine-readable form. The relevant accession numbers or links should be provided. When possible, standardized format (SBML, CellML) should be used instead of scripts (e.g. MATLAB). Authors are strongly encouraged to follow the MIRIAM guidelines (see link list at top right) and deposit their model in a public database such as Biomodels (see link list at top right) or JWS Online (see link list at top right). If computer source code is provided with the paper, it should be deposited in a public repository or included in supplementary information.	N/A

G- Dual use research of concern

22. Could your study fall under dual use research restrictions? Please check biosecurity documents (see link list at top right) and list of select agents and toxins (APHIS/CDC) (see link list at top right). According to our biosecurity guidelines, provide a statement only if it could.	N/A
